# Endoparasites of peritoneal organs and skeletal muscles of the European wildcat (*Felis silvestris*) in Germany

**DOI:** 10.1186/s13071-024-06571-4

**Published:** 2024-11-18

**Authors:** Katrin Bisterfeld, Marie-Kristin Raulf, Patrick Waindok, Andrea Springer, Johannes Lang, Michael Lierz, Ursula Siebert, Christina Strube

**Affiliations:** 1 Institute for Parasitology, Centre for Infection Medicine, University of Veterinary Medicine Hannover, Buenteweg 17, 30559 Hanover, Germany; 2 Institute for Terrestrial and Aquatic Wildlife Research, University of Veterinary Medicine Hannover, Werftstrasse 6, 25761 Buesum, Germany; 3 Clinic for Birds, Reptiles, Amphibians and Fish, Justus-Liebig-University Giessen, Frankfurter Strasse 114, 35392 Giessen, Germany

**Keywords:** Gastrointestinal parasites, Bladder worms, Liver flukes, Cestodes, *Echinococcus multilocularis*, Prevalence, Epidemiology

## Abstract

**Background:**

For several decades, the European wildcat (*Felis silvestris*) has gradually been returning to the forests of Germany, mainly in the central and southwestern regions. To increase the knowledge about this threatened species, the endoparasite status of dead found specimens from Germany was surveyed.

**Methods:**

A total of 118 wildcats were examined for endoparasites in peritoneal organs and skeletal muscles. Owing to decomposition or incomplete carcasses, 104 gastrointestinal tracts (stomachs and intestines), 101 livers with gallbladders, 99 urinary bladders, as well as kidneys of 95 and skeletal muscles of 112 specimens were available for examination. All detected parasites were identified morphologically to genus or species level, followed by molecular examinations of one to ten specimens of each parasite species.

**Results:**

Overall endoparasite prevalence in peritoneal organs was 99.0% (103/104). Among the 99.0% (103/104) infected gastrointestinal tracts, the most frequent species were *Toxocara cati* (95.2% [99/104]), *Hydatigera kamiyai* (84.6% [88/104]), *Mesocestoides litteratus* (69.2% [72/104]), *Strongyloides* spp. (58.7% [61/104]), *Cylicospirura petrowi* (37.5% [39/104]), *Ancylostoma tubaeforme* (31.7% [33/104]), *Capillaria putorii* (24.0% [25/104]), and *Echinococcus multilocularis* (18.3% [19/104]). In 77.8% (77/99) of the urinary bladders, *Capillaria plica* and/or *Capillaria feliscati* were detected. Moreover, the liver fluke *Metorchis bilis* occurred in 2.0% (2/101) of the livers, and roundworm larvae (presumably *Toxocara* spp.) were detected in 33.0% (37/112) of the muscle samples.

**Conclusions:**

These results show a broad spectrum of endoparasite species infecting European wildcats in Germany. It might be assumed that some of the endoparasites could pose a risk to domestic cats (*Felis catus*) and humans through spillover events, or may be transmitted from domestic cats to the free-ranging population, posing a potential risk to wildcats.

**Graphical Abstract:**

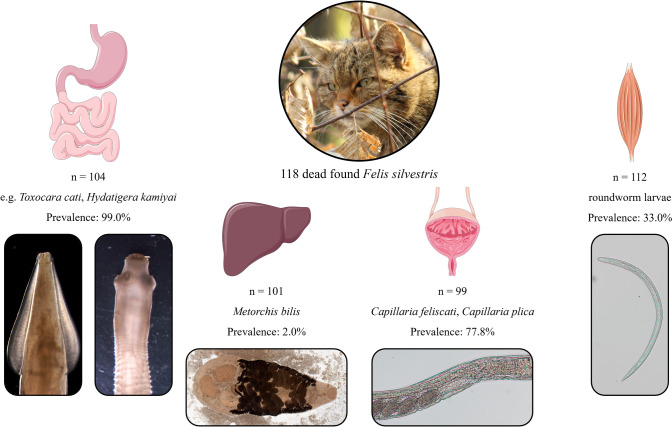

**Supplementary Information:**

The online version contains supplementary material available at 10.1186/s13071-024-06571-4.

## Background

Since European wildcats (*Felis silvestris*) are elusive and prefer structured deciduous and mixed forests as their natural habitat [1], sightings are rather rare. Although they mainly prey on rodents, they were heavily hunted in the early twentieth century owing to their reputation for preying on, e.g., hares (*Lepus europaeus*), pheasants (*Phasianus colchicus*), and roe deer (*Capreolus capreolus*) [2, 3]. Additional habitat fragmentation in the late twentieth century almost led to eradication of the species [2]. Today, wildcats are mainly endangered by loss of structure in their habitat and habitat fragmentation, as well as losses due to road accidents [1]. Nevertheless, this strictly protected species [4, 5] has spread again in Germany in recent years, especially in the north and west of the country [1, 6]. The German Federation for the Environment and Nature Conservation, Friends of the Earth Germany (Bund fuer Umwelt und Naturschutz Deutschland, BUND), has contributed to this success with numerous species protection projects. During one of these projects, infections with cardiopulmonary parasites and infestations with ectoparasites in wildcats found dead in the German federal state Rhineland-Palatinate were determined and published previously [7, 8].

The occurrence of other endoparasites in *F. silvestris* has been studied during this century in different European countries, such as Germany, Croatia, and Greece by necropsy of 15–77 carcasses [9–12]. The nematodes *Toxocara cati*, *Ancylostoma* spp., and *Cylicospirura* spp. and the cestodes *Hydatigera taeniaeformis* (syn. *Taenia taeniaeformis*) and *Mesocestoides* spp. were found in almost each of these studies and thus appear to be the most common gastrointestinal parasites of the European wildcat. The ascarid *T. cati* parasitizes in the small intestine of felids. In kittens, infections can cause gastrointestinal signs and emaciation [13]. *Ancylostoma tubaeforme* also resides in the small intestine of felines. As a hematophagous parasite, high infection rates in young or weakened animals can lead to anemia and weight loss [13, 14]. *Cylicospirura* spp. infect the stomachs of several felid species, where they cause formation of nodules in the gastric mucosa [14–16]. The species *Cylicospirura petrowi* is known to occur in German wildcats [12]. Of the common cestodes, *H. taeniaeformis* is similarly prevalent in *F. silvestris* as *T. cati* [9–12]. The adult worms located in the small intestine can grow up to 60 cm in length, and severe infection may lead to gastrointestinal obstruction [13, 14]. *Mesocestoides* spp. also live in the small intestine but are much smaller (4–14 cm long) [17].

Studies on wildcats’ endoparasites in peritoneal organs other than the gastrointestinal tract are less frequent, resulting in a paucity of data. However, the urinary bladder worms *Capillaria plica* (syn. *Pearsonema plica*) and *Capillaria feliscati* (syn. *Pearsonema feliscati*) have previously been detected in *F. silvestris* in Germany [10, 12].

As *F. silvestris* is classified as a Threatened species in Germany’s Red List [18], which equals Vulnerable on the IUCN Red List, it is important to gather data about its parasitological and general health status. Therefore, the aim of this study was to provide current data on prevalence and infection intensity of endoparasites in peritoneal organs and skeletal muscles of wildcats in Germany.

## Methods

### Sample material

**Fig. 1:**
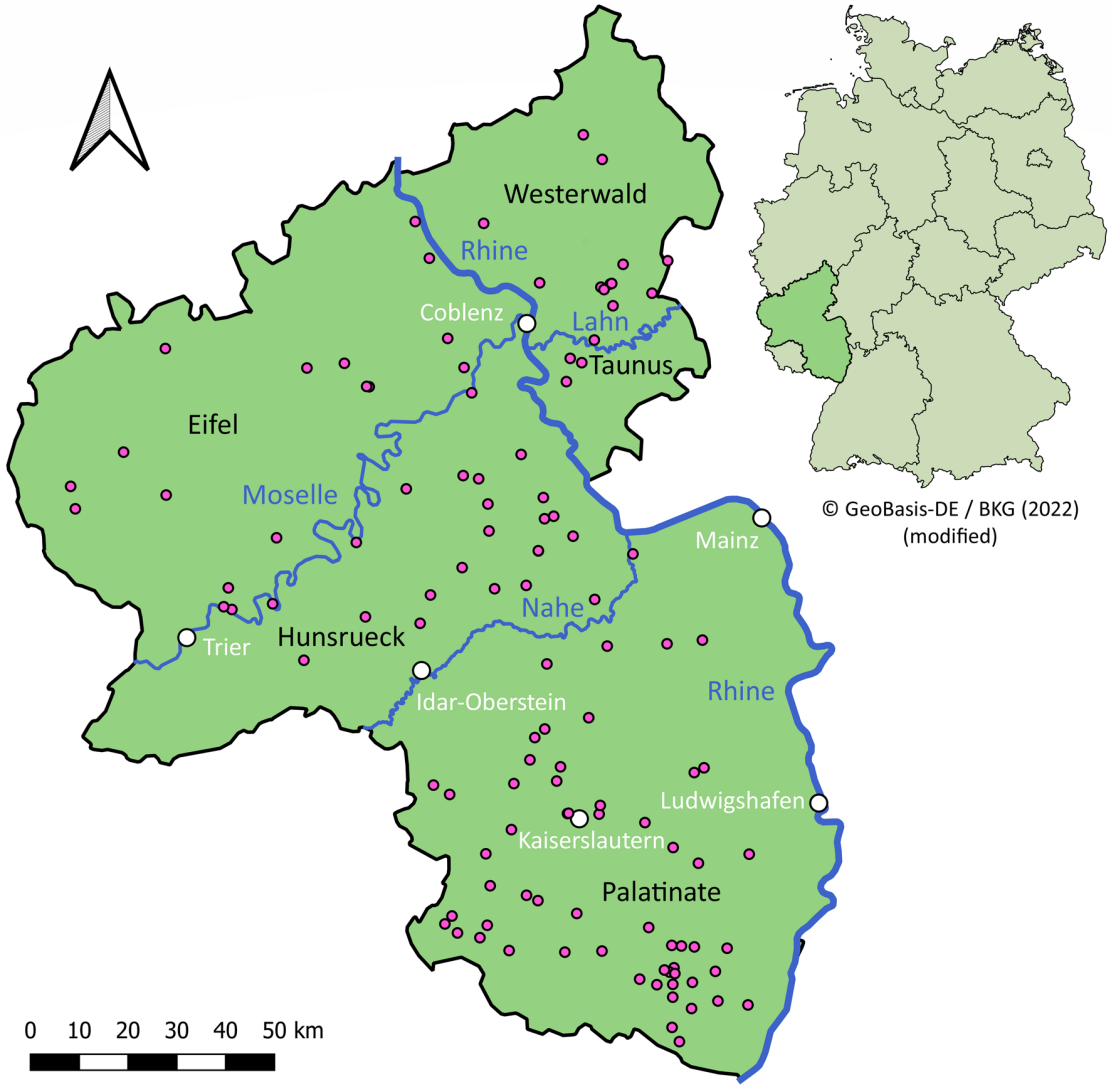
Map of the German federal state Rhineland-Palatinate, showing the origin of the examined wildcats (pink dots). Scale bar = 50 km. Arrow points north. The map was modified from Bisterfeld et al. [7] using QGIS version 3.22.2 [66]. The map of Germany (insert) was obtained from GeoBasis-DE/BKG [67]

All wildcat samples were gathered in the frame of the project Monitoring of dead wildcats in Rhineland-Palatinate (*Totfundmonitoring Wildkatze in Rheinland-Pfalz*) of the German Federation for the Environment and Nature Conservation, Friends of the Earth Germany, state association of Rhineland-Palatinate [Bund fuer Umwelt und Naturschutz Deutschland (BUND), Landesverband Rheinland-Pfalz]. Dissections of dead found specimens and determination of parameters such as species, nutritional condition, age, sex, and state of decomposition were performed by BUND and the cooperating institutions Will and Liselott Masgeik Foundation, OEKOLOG field research, and the Working group of the Group for Wildlife Research of the Clinic for Birds, Reptiles, Amphibians, and Fish, Justus-Liebig-University Giessen, Germany [7, 19]. Species diagnosis as *F. silvestris* was carried out morphometrically and genetically using reduced single-nucleotide polymorphism panels [20] as previously described [7, 19]. The categorization of the nutritional condition was based on subcutaneous, visceral, coronary, and kidney fat deposits as described in Bisterfeld et al. [7]. All 118 animals examined were collected in the German state of Rhineland-Palatinate between 2018 and 2020. Figure 1 shows the specific locations of their collection in the regions of Palatinate, Hunsrueck, Westerwald, Eifel, and Taunus. The material for examination was stored at −20 °C and transferred to the University of Veterinary Medicine Hannover, Germany. Before starting parasitological investigations, all intestines were frozen at −80 °C for at least 48 h, as recommended by the WOAH [21], to avoid the risk of zoonotic infections, especially with *Echinococcus multilocularis*.

### Parasitological examinations

#### Gastrointestinal tract including liver

**Fig. 2:**
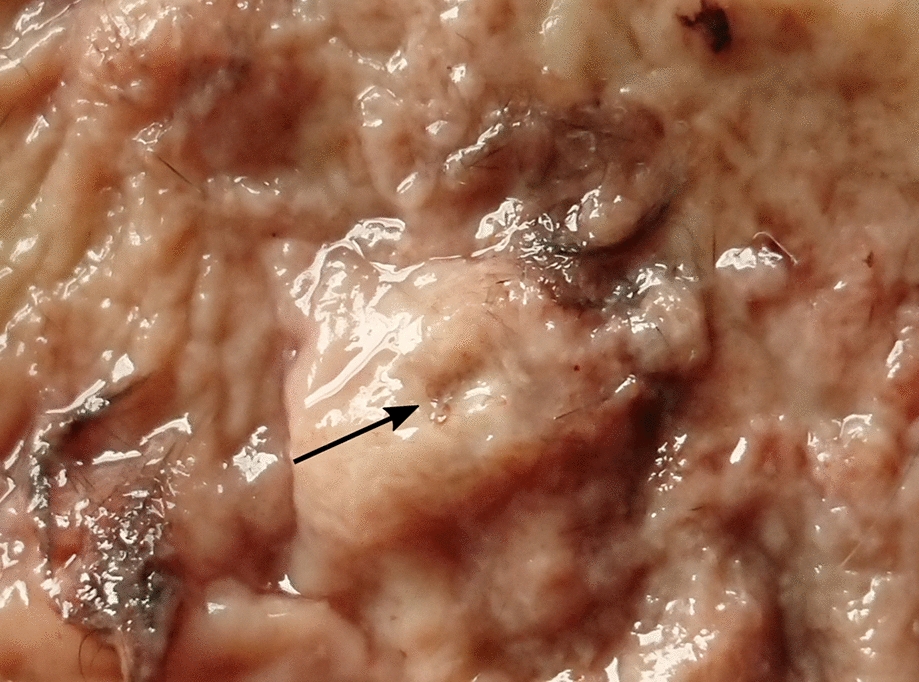
Gastric nodule with central opening (arrow) and protruding specimens of *Cylicospirura petrowi* in *Felis silvestris*

The examination of the stomachs was performed using a modified methodology described by Waap et al. [22]: The stomachs were opened along the great curvature, and contents were removed and examined macroscopically for parasites. The mucosa was then screened for nodules (Fig. 2). Existing nodules were counted and opened to extract nematodes. Subsequently, the whole mucosa was scraped with a scalpel and transferred into a 50-ml tube containing 30–45 ml denatured ethanol (70%, Carl Roth GmbH + Co. KG, Karlsruhe, Germany). After vigorous shaking, the content was flushed through a 150-µm sieve, and the sieve residues were examined at 8–12× magnification (stereomicroscope SLX-2; OPTIKA S.r.l., Ponteranica, Italy).

After thawing the intestines, feces were removed from the rectum. Then, the small and large intestines were separated, opened lengthwise, and incubated for 30 min at 37 °C in physiological saline solution (0.9% NaCl). After stripping the entire mucosa between the fingers and removal of coarse hairy fecal bales, the small intestinal contents were flushed with tap water through three sieves (mesh sizes: top 500 µm, middle 100 µm, bottom 50 µm), and the large intestinal contents through a single 50-µm sieve. The individual sieve contents were then examined at 8–15× magnification (stereomicroscope SLX-2; OPTIKA S.r.l., Ponteranica, Italy). If the contents of the 100-µm and 50-µm sieves amounted to more than 30 ml, an aliquot of 10–20 ml was examined and the results were extrapolated accordingly, while the contents of the 500-µm sieves were always examined completely.

The livers were sliced, and the gallbladder and bile ducts opened with knifes or scissors and examined at 7× magnification under a stereomicroscope (SLX-2; OPTIKA S.r.l., Ponteranica, Italy).

#### Urinary system

The kidneys were sliced with a knife, and renal pelvises were examined macroscopically for the presence of nematodes. The urinary bladders were opened with scissors, and the urine collected. According to Takeuchi-Storm et al. [23], the whole bladder mucosa was scraped with a scalpel blade and additionally washed with physiological saline solution (0.9% NaCl). Then, mucosa scrapings and wash as well as the urine were examined at 10–15× magnification (stereomicroscope SLX-2; OPTIKA S.r.l., Ponteranica, Italy).

#### Skeletal muscles

The examination of the musculature was carried out following the official examination for *Trichinella* as described in the Commission Implementing Regulation (EU) 2015/1375 [24]. Pieces of mean 23.7 g muscles (0.9–55.9 g; median 29.3 g) were homogenized with a hand blender in at least 200 ml digestion solution containing 10 ml HCl (37% [Carl Roth GmbH + Co. KG, Karlsruhe, Germany]) and 10 g pepsin (≥ 2000 FIP-U/g [Carl Roth GmbH + Co. KG, Karlsruhe, Germany]) per liter. Digestion was carried out at 37 °C for 30–45 min with constant stirring. After the muscle pieces were dissolved, the digestion solution was rinsed through two sieves (mesh sizes: top 500 µm, bottom 50 µm) to separate coarse undigested pieces. The contents of the 50-µm sieve were recovered and examined at 40× magnification (Primostar 1; Carl Zeiss GmbH, Vienna, Austria).

All parasites recovered from the different organs were determined by morphological characteristics according to published descriptions [12–14, 25–38].

#### Molecular species determination

The morphological diagnoses of 1–10 specimens per endoparasite species were confirmed by molecular analyses. DNA extraction was performed as described by Bisterfeld et al. [7] using 90 μl DirectPCR lysis reagent (DirectPCR^®^ cell lysis reagent; Viagen Biotech Inc., Los Angeles, CA, USA) and 10 μl Proteinase K (20 mg/ml; Macherey–Nagel GmbH & Co. KG, Dueren, Germany) for lysis of larger parasites, e.g., *T. cati*, *Hydatigera kamiyai*, and *Metorchis bilis* or half of each volume for smaller species such as *Strongyloides* spp. and *Capillaria* spp. and muscle larvae. After disruption with a pestle, incubation for 14–16 h at 55 °C was followed by inactivation of Proteinase K for 45 min at 85 °C [7].

Multiplex PCR was performed to amplify the NADH dehydrogenase subunit 1 (NAD-1) of *E. multilocularis* and the small subunit of ribosomal RNA (SSU rRNA) of *Mesocestoides litteratus*, *Taenia martis*, and *H. kamiyai* [39].

Singleplex amplification of the NAD-1 region of *H. kamiyai* [40], the internal transcribed spacer (ITS) region (ITS1-5.8S rRNA-ITS2) of *T. cati*, *Strongyloides* spp., *A. tubaeforme*, *Molineus* spp., *Syphacia frederici*, *Heligmosomum* spp., and *Porrocaecum* spp. [41], and the cytochrome c oxidase subunit 1 (COX-1) of *Capillaria* spp., *C. petrowi*, and *M. bilis* [15, 42, 43] were performed as follows: For a 25 µl PCR reaction volume, 0.5 µl DreamTaq DNA polymerase (5.0 U/µl), 2.5 µl 10 × DreamTaq buffer (Thermo Fisher Scientific Inc., Waltham, MA, USA), and 0.5 µl dNTPs (10 mM each, Roti^®^-Mix PCR 3, Carl Roth GmbH + Co. KG, Karlsruhe, Germany) were used. For 50 µl reaction volumes, the volume of DreamTaq DNA polymerase was halved and the volume of DreamTaq buffer and dNTPs was doubled. Detailed information on primers, template volumes, and cycling conditions used for molecular analyses of the different endoparasite species is listed in Table 1.

**Table 1:** Target regions, primers, and cycling conditions used for molecular analyses of different endoparasite species of European wildcats

Species	DNA region	Primers	Amplicon size	Reaction volume/template	Temperature profile	Ref.
*E. multilocularis*	NAD-1	Cest1, Cest2, Cest3, Cest4 (2 µM each), Cest5 (16 µM)	400 bp	25 µl/5 µl	95 °C for 15 min; 40 × 94 °C for 30 s, 58 °C for 90 s, 72 °C for 10 s	[39]
*M. litteratus*	SSU rRNA		280 bp	25 µl/5 µl		
*T. martis*			250 bp	25 µl/5 µl		
*H. kamiyai*			250 bp	25 µl/5 µl		
*H. kamiyai*	NAD-1	nad1T-Fw, nad1T-Rv (0.2 µM each)	500 bp	25 µl/1 µl	95 °C for 3 min; 45 × 94 °C for 30 s, 52 °C for 30 s, 72 °C for 60 s; 72 °C for 10 min	[40]
*T. cati*	ITS1-5.8S rRNA-ITS2	NC5, NC2 (0.2 µM each)	~ 1100 bp	25 µl/5 µl	95 °C for 3 min; 40 × 94 °C for 30 s, 55°C^1^ for 30 s, 72 °C for 90 s; 72 °C for 10 min	[41]
*Strongyloides* spp.			~ 550 bp	25 µl/2–5 µl		
*A. tubaeforme*			~ 850 bp	25 µl/1 µl		
*Molineus* spp.			~ 850 bp	25 µl/3 µl		
*S. frederici*			~ 850 bp	50 µl/5 µl		
*Heligmosomum* spp.			~ 100 bp	25 µl/5 µl		
*Porrocaecum* spp.			~ 1200 bp	25 µl/5 µl		
*C. putorii*	COX-1 mtDNA	COIFmod, COIRmod (1.0 µM each)	400 bp	25 µl/5 µl	95 °C for 3 min; 40 × 94 °C for 30 s, 54 °C for 60 s, 72 °C for 60 s; 72 °C for 10 min	[42]
*C. plica*/*C. feliscati*			400 bp	50 µl/5 µl		
*C. plica*/*C. feliscati*		COICapillF2, COICapillR2 (1.0 µM each)	250 bp	25 µl/5 µl	95 °C for 3 min; 40 × 94 °C for 30 s, 55 °C for 60 s, 72 °C for 60 s; 72 °C for 10 min	
*C. petrowi*	COX-1 mtDNA	NTF, NTR (0.5 µM each)	700 bp	50 µl / 5 µl	95 °C for 5 min; 40 × 95 °C for 30 s, 50 °C for 60 s, 72 °C for 60 s; 72 °C for 7 min	[15]
*M. bilis*	COX-1 mtDNA	CO1-F Plagi, CO1-R Plagi (0.8 µM each)	~ 490 bp	50 µl / 1 µl	95 °C for 3 min; 35 × 95 °C for 30 s, 55 °C for 30 s, 72 °C for 60 s; 72 °C for 7 min	[43]

^1^ Annealing temperature of 50 °C for *Strongyloides* spp

Visualization of DNA amplicons was achieved using 1.5% or 2.0% agarose gel supplemented with GelRed^®^ (1:10,000; Biotium, Inc., Fremont, CA, USA), and bands of the expected size were custom Sanger sequenced (Microsynth Seqlab GmbH, Goettingen, Germany). Sequences were reviewed and compared using the Clone Manager software (version 9 professional, Sci-Ed, Westminster, Colorado, USA) and BLASTed against the NCBI standard databases. Obtained sequences with good quality were deposited in NCBI GenBank under accession numbers PP796350 (*E. multilocularis*), PP808785–PP808790 (*T. martis*), PP808783–PP808784 (*H. kamiyai*, SSU rRNA), PP796351–PP796355 (*H. kamiyai*, NAD-1), PP808791–PP808800 (*M. litteratus*), PP812129–PP812131 (*T. cati*), PP812123 (*A. tubaeforme*), PP812124–PP812125 (*Molineus* spp.), PP812126–PP812128 (*Strongyloides* spp.), PP840086 (*C. petrowi*), PP840083–PP840085 (*Capillaria putorii*, syn. *Aonchotheca putorii*), PP840087–PP840090 (*C. feliscati*), PP840091–PP840097 (*C. plica*), and PP840081–PP840082 (*M. bilis*).

### Statistical analyses

Statistical analyses were performed using R version 4.1.0 [44]. The 95% confidence intervals for prevalence values were calculated using the R function “binom.test.” The effects of the predictor variables “sex,” “age,” “nutritional status,” “season of finding,” and “state of decomposition” on the prevalence of *E. multilocularis*, *M. litteratus*, *H. kamiyai*, *Strongyloides* spp., *A. tubaeforme*, *C. putorii*, *C. plica*, *C. feliscati*, and *C. petrowi* were calculated using generalized linear models (GLMs) with binominal error structure. The factors “very good” and “good” of the predictor variable “nutritional condition” were summarized for a suitable analysis. No calculations were made for the remaining parasite species because of either a very high or very low prevalence.

In an additional GLM with Poisson error structure and log link function, the effects of the above-mentioned predictors on endoparasite species richness (i.e., the sum of co-infections with gastrointestinal parasites [*E. multilocularis*, *M. litteratus*, *H. taeniaeformis*, *T. martis*, *T. cati*, *Strongyloides* spp., *A. tubaeforme*, *Molineus* spp., *Physaloptera* spp., *C. putorii*, and *C. petrowi*] and urinary parasites [*C. plica* and *C. feliscati*]) were investigated. Owing to missing data, the models were based on 75 animals for gastrointestinal and 71 animals for urinary parasites, respectively.

For the factor “season of finding,” multiple comparisons were calculated using Tukey contrasts with single-step *P* value adjustment. To test the validity of the GLMs, each model was compared with a null model containing only the intercept using a likelihood ratio test.

## Results

### Collected wildcat specimens

A total of 118 different specimens were examined, all of which were identified as *F. silvestris*. Owing to advanced decomposition or missing organs, the available examination material included the gastrointestinal tract (stomachs and intestines) of 104, livers with gallbladders of 101, urinary bladders of 99, kidneys of 95, and skeletal muscles of 112 different wildcats.

All wildcat carcasses were collected in the German federal state of Rhineland-Palatinate, in the regions Palatinate (49.2%), Hunsrueck (18.6%), Eifel (15.3%), Westerwald (11.9%), and Taunus (2.5%). Sex determination was possible for 61 (51.7%) males and 42 (35.6%) females. The sex of the remaining 15 (12.7%) specimens could not be identified because the carcasses were too rotten or incomplete [19]. Most of the wildcats were adult (43.2%), and the most common nutritional conditions were good or very good (68.6%). Most specimens were found in autumn (September, October, November; 45.8%), followed by winter (December, January, February; 20.3%), spring (March, April, May; 19.5%), and summer (June, July, August; 13.6%). The carcasses were mainly fresh (43.2%) or moderately fresh (44.9%), and in 94.1% of all cases, the cause of death was suspected to be trauma, mostly caused by road traffic. Table 2 shows the key data in detail.

**Table 2:** Key data of the examined wildcats (*n* = 118)

Parameter	Number of wildcats (%)
Sex	
Male	61 (51.7)
Female	42 (35.6)
Not determinable	15 (12.7)
Age	
Adult (> 25 months)	51 (43.2)
Subadult (11–24 months)	18 (15.3)
Immature (5–10 months)	23 (19.5)
Juvenile (< 4 months)	5 (4.2)
Not determinable	21 (17.8)
Nutritional condition	
Very good	40 (33.9)
Good	41 (34.7)
Moderate	18 (15.3)
Very bad/cachectic	10 (8.5)
Not determinable	9 (7.6)
Season of finding	
Spring	23 (19.5)
Summer	16 (13.6)
Autumn	54 (45.8)
Winter	24 (20.3)
Not determinable	1 (0.8)
State of decomposition	
Fresh	51 (43.2)
Moderately fresh	53 (44.9)
Moderately rotten	11 (9.3)
Proceeded rotten	3 (2.5)
(Suspected) cause of death	
Trauma	111 (94.1)
Cachexia	1 (0.8)
Diarrhea	1 (0.8)
Unknown	5 (4.2)

Additional file 1 summarizes the key data and parasitological results of individual wildcats from which at least three different organs were examined, including previously published data on cardiopulmonary parasites [7] and ectoparasites [8].

### Gastrointestinal and hepatic parasites

In the gastrointestinal tracts of the different wildcats, endoparasites were detected in all but one juvenile wildcat. In more detail, endoparasites were found in 85/104 stomachs (81.7%) and 103/104 intestines (99.0%).

**Fig. 3:**
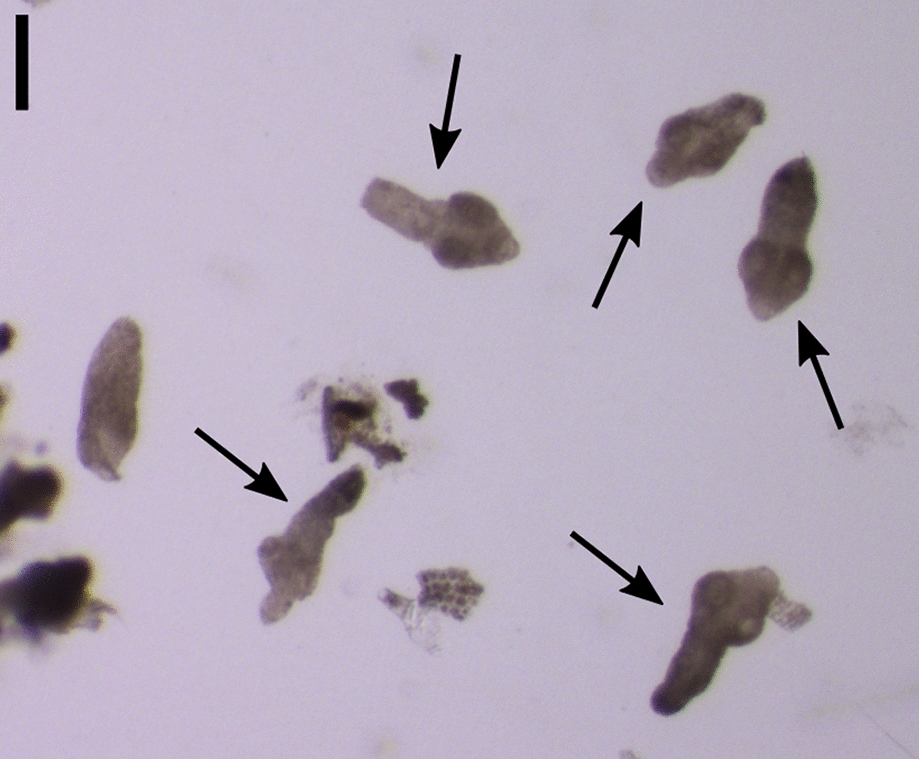
Specimens of *Echinococcus multilocularis* (arrows) detected in *Felis silvestris*. Scale bar represents 200 µm

**Fig. 4:**
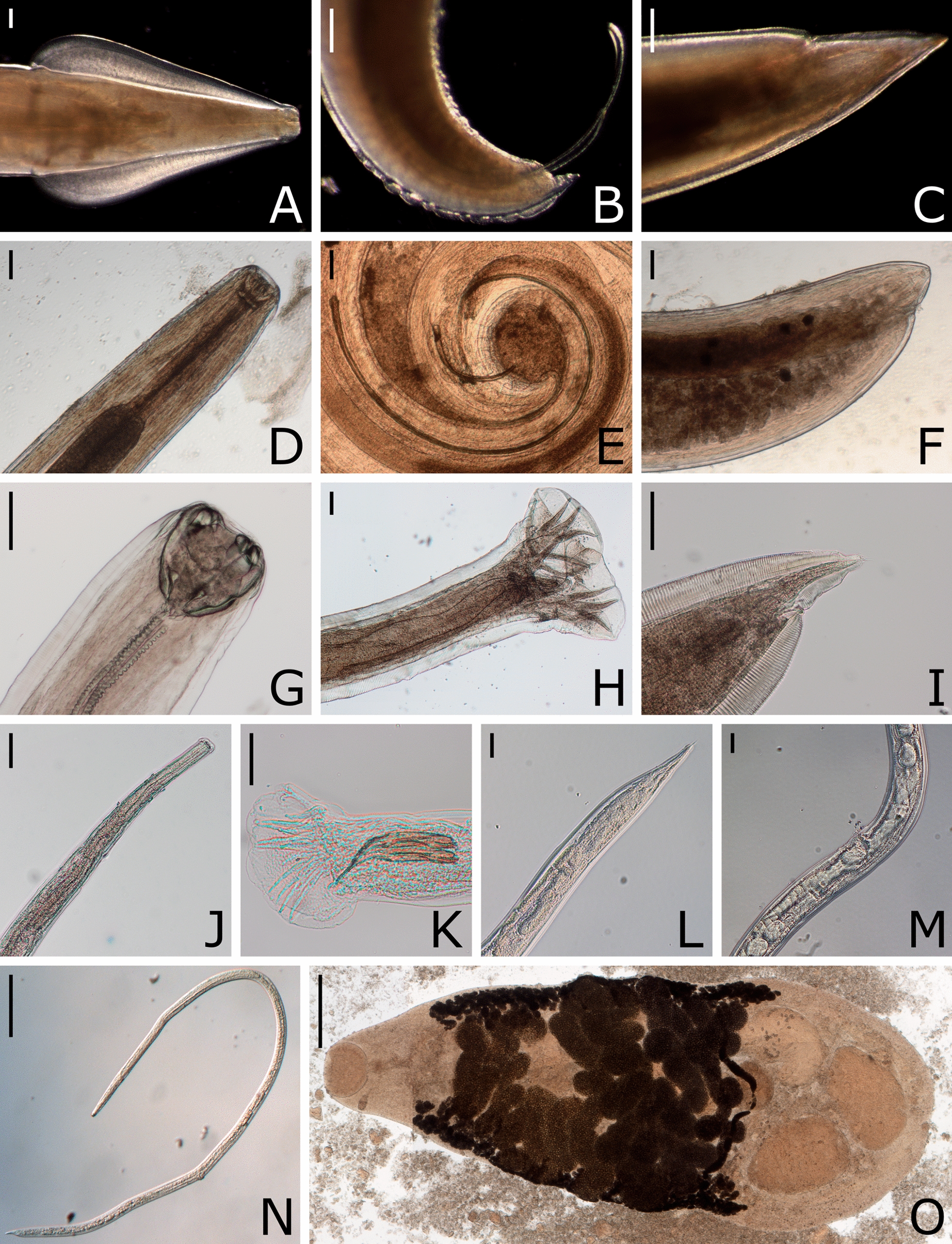
Endoparasites detected in the gastrointestinal tract or gallbladders of *Felis silvestris*. Representative specimens of *Toxocara cati* (**A–C**; **A** anterior end, **B** male posterior end, **C** female posterior end; scale bars represent 200 µm), *Cylicospirura petrowi* (**D-F**; **D** anterior end, **E** male posterior end, **F** female posterior end; scale bars represent 100 µm), *Ancylostoma tubaeforme* (**G-I**; **G** anterior end, **H** male posterior end, **I**: female posterior end; scale bars represent 100 µm), *Molineus* spp. (**J-M**; **J** anterior end, **K** male posterior end, **L** female posterior end, **M** vulva region; scale bars represent 50 µm), female *Strongyloides* spp. (**N**; scale bar represents 200 µm), *Metorchis bilis* (**O**; scale bar represents 500 µm)

**Fig. 5:**
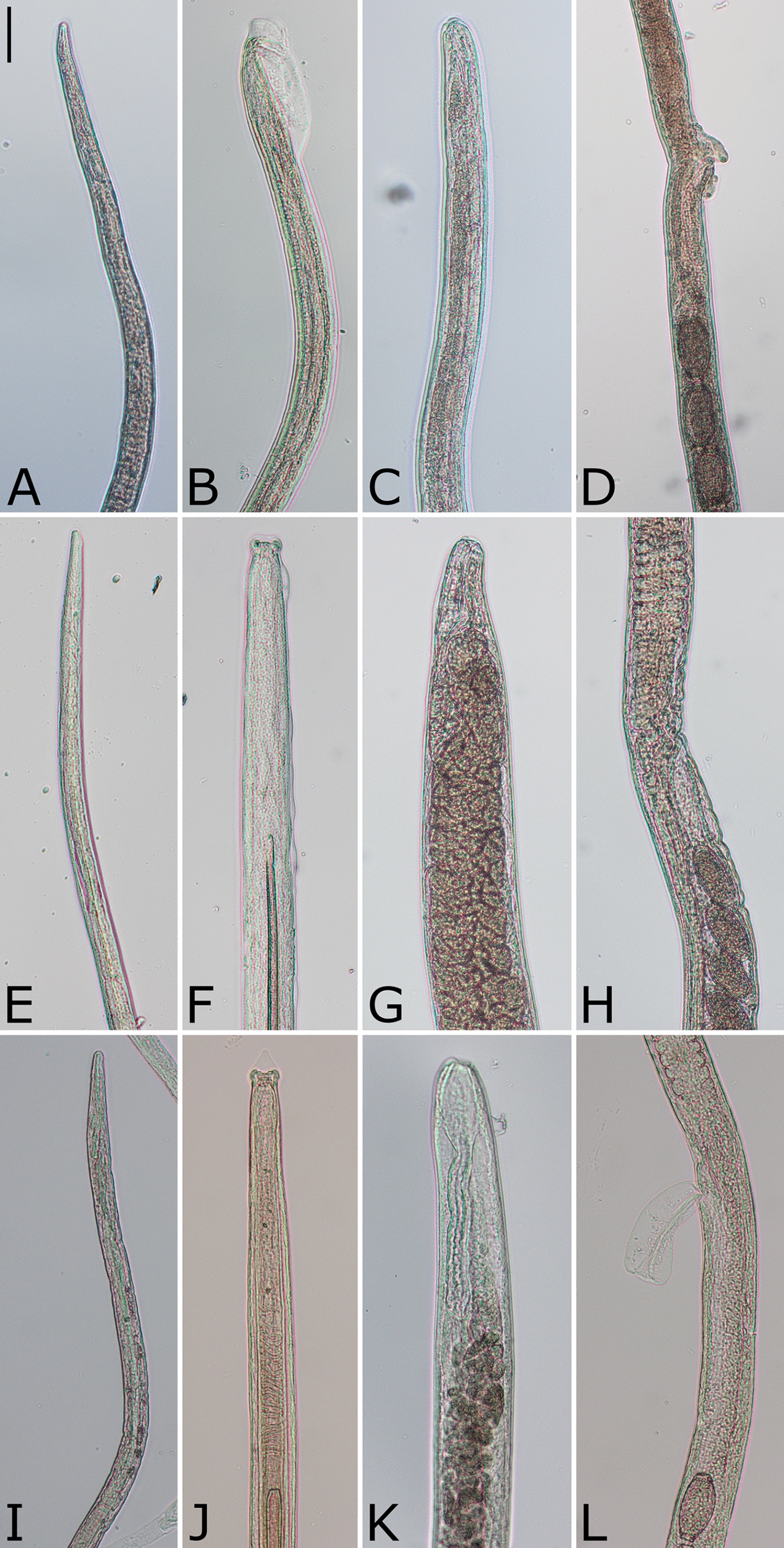
Different *Capillaria* species detected in the gastrointestinal tract or urinary bladders of *Felis silvestris*. Representative specimens of *Capillaria putorii* (**A–D**; **A** anterior end, **B** male posterior end, **C** female posterior end, **D** vulva region), *Capillaria feliscati* (**E–H**; **E** anterior end, **F** male posterior end, **G**: female posterior end, **H** vulva region), *Capillaria plica* (**I–L**; **I** anterior end, **J** male posterior end, **K** female posterior end, **L** vulva region). Scale bar represents 50 µm

**Fig. 6:**
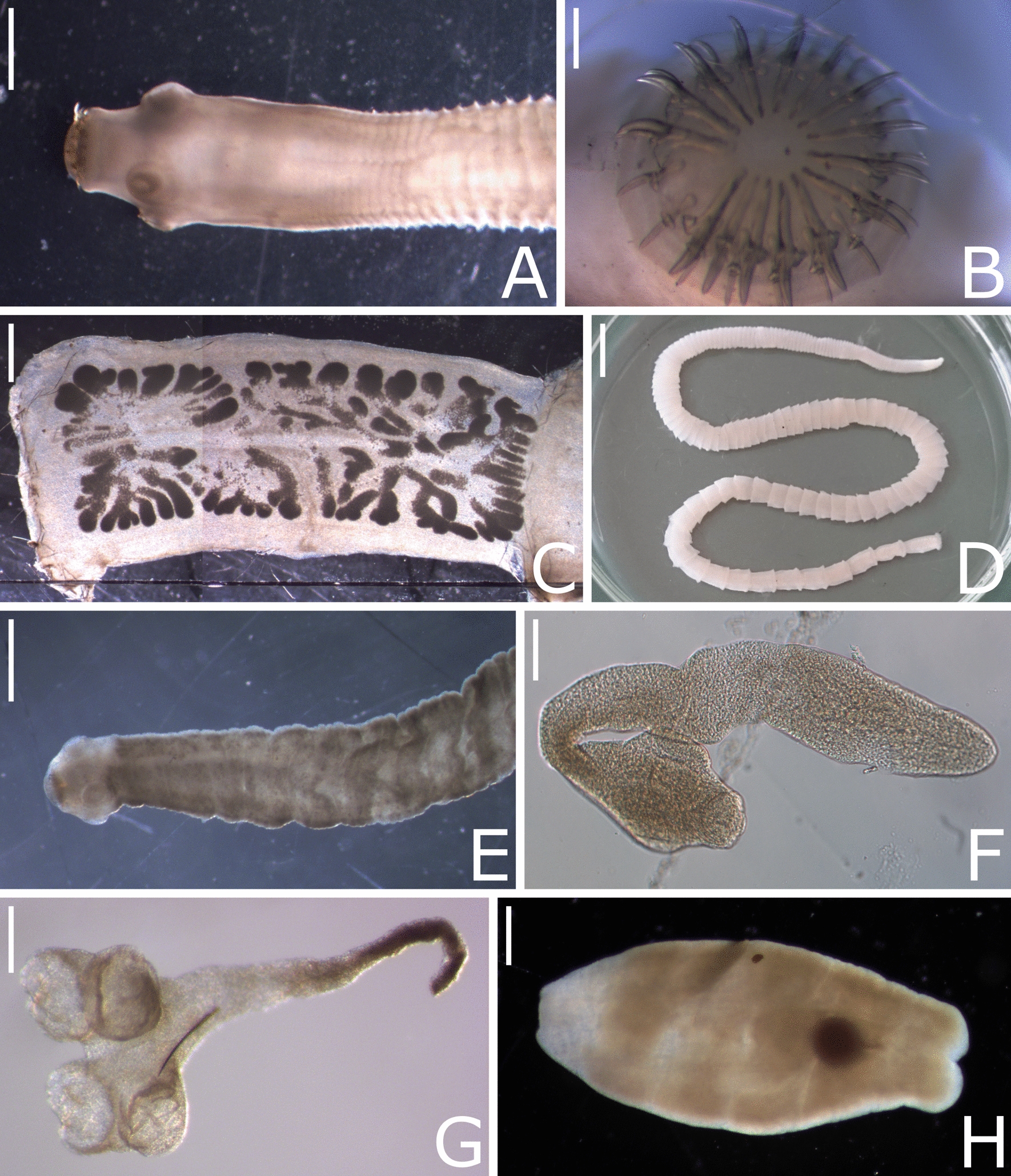
Cestodes detected in the gastrointestinal tract of *Felis silvestris*. Representative specimens of *Hydatigera kamiyai* (**A–D**; **A** anterior end with scolex, scale bar represents 1 mm, **B** rostellar hooks, scale bar represents 200 µm, **C** gravid proglottid, scale bar represents 1 mm, **D** whole specimen, scale bar represents 1 cm), *Taenia martis* (**E** anterior end with scolex, scale bar represents 1 mm), *Echinococcus multilocularis* (**F** whole specimen, scale bar represents 100 µm), *Mesocestoides litteratus* (**G–H**; **G** anterior end with scolex, scale bar represents 200 µm, **H** gravid proglottid, scale bar represents 500 µm)

In 42 (40.4%) of the stomachs, 1–12 (mean 2.1; median 1.0) nodules of *C. petrowi* (Fig. 2) were present. Specimens of *C. petrowi* were found in 36 (85.7%) of these stomachs as well as in three stomachs without detectable nodules, amounting to a total parasite prevalence of 37.5% (39/104). In six additional stomachs, 1–5 nodules were present, but no *C. petrowi* specimens were found. Furthermore, one *Physaloptera* sp. individual was detected in the stomach of one animal (1.0%). *Toxocara cati* (95.2%), *H. kamiyai* (84.6%), *Strongyloides* spp. (58.7%), and *C. putorii* (24.0%) were found in both intestines and stomachs, while *Mesocestoides litteratus* (69.2%), *A. tubaeforme* (31.7%), *E. multilocularis* (18.3%), *T. martis* (9.6%), and *Molineus* spp. (5.8%) were present in the intestines only. Interestingly, all detected *E. multilocularis* specimens appeared to be immature and did not show gravid proglottids (Fig. 3). Exemplary photographs of detected endoparasites are shown in Figs. 4, 5, and 6, and detailed information on prevalence is given in Table 3.

**Table 3:** Prevalence and infection intensity of endoparasites detected in European wildcats in Germany

Parasite	Positive wildcats	Prevalence (%)	95% confidence interval (%)	Infection intensity			
				Min.	Max.	*Ø*	Median
Gastrointestinal tract (*n* = 104)							
*Toxocara cati* ^i+s^	99	95.2	89.1–98.4	1	411	59.9	39.0
*T. cati* ^i^	97	93.3	86.6–97.3	1	260	53.2	34.0
*T. cati* ^s^	71	68.3	58.4–77.1	1	186	11.0	4.0
*Hydatigera kamiyai* ^i+s^	88	84.6	76.2–90.9	1	53	7.4	4.5
*H. kamiyai* ^i^	88	84.6	76.2–90.9	1	52	7.2	4.0
*H. kamiyai* ^s^	8	7.7	3.4–14.6	1	5	1.9	1.0
*Mesocestoides litteratus* ^i^	72	69.2	59.4–77.9	1	3926	225.1	74.0
*Strongyloides* spp. ^i+s^	61	58.7	48.6–68.2	4	2400	164.3	36.0
*Strongyloides* spp. ^i^	61	58.7	48.6–68.2	4	2400	164.3	36.0
*Strongyloides* spp. ^s^	1	1.0	0.0–5.2	2	2	2.0	2.0
*Cylicospirura petrowi* ^s^	39	37.5	28.2–47.5	1	37	7.4	5.0
*Ancylostoma tubaeforme* ^i^	33	31.7	22.9–41.6	1	135	13.0	2.0
*Capillaria putorii* ^i+s^	25	24.0	16.2–33.4	1	389	21.2	1.0
*C. putorii* ^i^	2	1.9	0.2–6.8	6	31	18.5	18.5
*C. putorii* ^s^	24	23.1	15.4–32.4	1	358	20.5	1.0
*Echinococcus multilocularis* ^i^	19	18.3	11.4–27.1	14	7506	965.3	322.0
*Taenia martis* ^i^	10	9.6	4.7–17.0	1	5	1.6	1.0
*Molineus* spp. ^i^	6	5.8	2.1–12.1	2	90	35.8	24.0
*Physaloptera* spp. ^s^	1	1.0	0.0–5.2	1	1	1.0	1.0
Unknown ^i^	3	2.9	0.6–8.2	2	75	28.0	7.0
Total	103	99.0	94.8–100	2	7597	512.4	208.0
Intestines	103	99.0	94.8–100	2	7594	496.7	181.0
Stomachs	85	81.7	72.9–88.6	1	475	18.6	6.0
Urinary bladders (*n* = 99)							
*Capillaria feliscati*/*plica*	77	77.8	68.3–85.5	1	75	7.0	4.0
*C. feliscati*	34	34.3	25.1–44.6	1	12	2.8	2.0
*C. plica*	28	28.3	19.7–38.2	1	24	3.0	2.0
*Capillaria* spp.^1^	68	68.7	58.6–77.6	1	59	5.3	3.0
Gall Bladders (*n* = 101)							
*Metorchis bilis*	2	2.0	0.2–7.0	1	6	3.5	3.5
Skeletal muscles (*n* = 112)							
Roundworm larvae (presumably *Toxocara* spp.)	37	33.0	24.4–42.6	0.3^2^	16.7^2^	2.3^2^	0.9^2^

^i^ in the intestines; ^s^ in the stomachs; ^1^ all cats with undeterminable specimens; ^2^ larvae in 10 g skeletal muscles

Apart from one juvenile wildcat, in which no endoparasites were found, only three animals were found to be monoinfected, each of which harbored *T. cati* (3/104, 2.9%). All other wildcats (100/104, 96.2%) were coinfected with up to eight different gastrointestinal helminth species. Most of the coinfected wildcats (90.0%) were infected with 3–6 different species (21–24 wildcats each), while only 10.0% of wildcats harbored two, seven, or eight different helminth species (1–5 wildcats each).

**Fig. 7:**
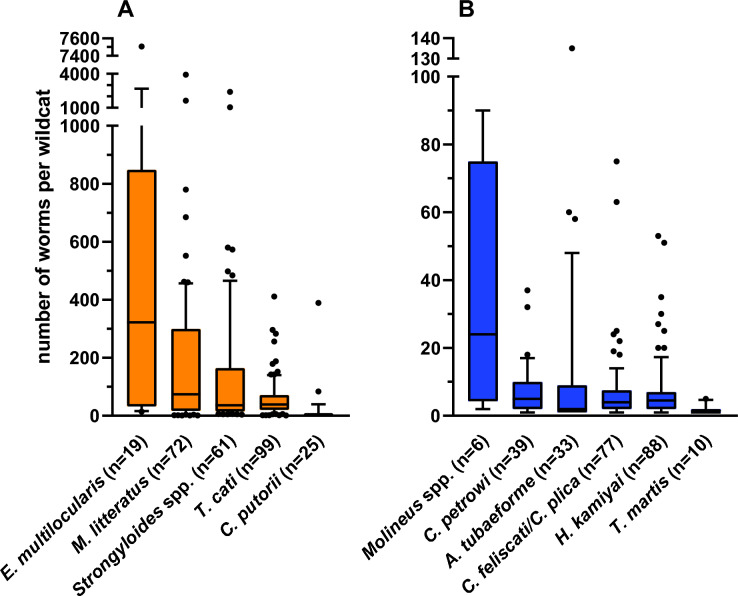
Infection intensities of the most prevalent endoparasites of *Felis silvestris*, divided into higher (**A**) and lower (**B**) levels of intensity (*n* = number of infected wildcats). The boxes end at the 25th and 75th percentiles, with a line at the median. The whiskers end at the 10th and 90th percentiles, and dots represent outliers

The infection intensity with *T. cati* amounted to 1–411 specimens per wildcat. Most (63.6%) of the 99 *T. cati*-infected wildcats harbored 1–50 worms, followed by intensities between 50 and 100 and more than 100, each with 18.2%. The intensity of *H. kamiyai* was 1–53 specimens in the 88 infected wildcats. Only 17.0% of the wildcats were infected with ten or more worms, while the remaining 82.9% harbored fewer than 10 scolices. The number of *A. tubaeforme* amounted to 1–135 worms per wildcat. The majority (75.8%) of the 33 positive animals showed infections with only up to five hookworms, while 21.2% of the wildcats harbored 13–60, and one animal (3.0%) 135 specimens. The intensity of the 25 *C. putorii*-infected wildcats ranged between 1 and 389 worms. However, most wildcats (92.0%) harbored 1–10 worms, while only two animals (8.0%) presented with 84 and 389 worms, respectively. Detailed information on infection intensities is listed in Table 3, while Fig. 7 displays the values of the most prevalent gastrointestinal helminths graphically.

In addition, spurious parasites, i.e., passengers originating from prey animals, were detected in the wildcats´ gastrointestinal tracts. In five wildcats, 1–20 *S. frederici* (Ø 6.4; median 3.0) were identified, in four wildcats 1–2 *Heligmosomum* spp. (Ø 1.5; median 1.5), and in one wildcat 17 *Porrocaecum* spp. specimens. Furthermore, one *Uncinaria* sp. individual was detected in the stomach of one wildcat, and one *Trichuris* sp. individual in another.

Molecular analyses allowed determination or confirmation of most gastrointestinal parasite genera/species based on up to ten analyzed individuals each. The nucleotide identity to respective sequences published in GenBank was 99.9–100% for *T. cati* (query cover [QC]: 99–100%; acc. nos. KY003066, KY003069). For *H. kamiyai*, the identity was 98.5–99.8% (QC 100%; acc. nos. JQ663994 [*T. taeniaeformis*], NC_037071 [*H. kamiyai*]) for the NAD-1 amplicon and 100% (QC 100%; acc. nos. MN505198, OQ834418-OQ834425 [*H. kamiyai*]) for the SSU rRNA amplicons. The SSU rRNA sequences were also 100% identical to a *H. taeniaeformis* sequence (QC 100%; acc. no. OP646456) but differed at 9.1–11.0% from two other *H. taeniaeformis* sequences (QC 100%; acc. nos. OR790451, MK779011). The obtained sequence of *M. litteratus* showed 99.0–100% identity (QC 100%; acc. nos. MN505203, MN505210). Further identities were 100% for *C. petrowi* (QC 100%; acc. no. KF719952), 99.0% for *A. tubaeforme* (QC 99.6%; acc. no. JQ812691), 98.3–100% for *C. putorii* (QC 100%; acc. no. KC355429), 98.7% for *E. multilocularis* (QC 100%; acc. no. MN444806), 100% for *T. martis* (QC 100%; acc. nos. AB731758, JX415820), and 98.8% for *S. frederici* (QC 100%; acc. no. MN652172).

Molecular analysis of *Strongyloides* spp. yielded no highly similar matches with sequences available in GenBank. All three sequences generated in the present study were 100% identical to each other, but only 85.7% (QC 88%) to *Strongyloides callosciureus* (acc. no. AB272229), 86.41% (QC 75%) to *Strongyloides fuelleborni fuelleborni* (acc. no. AB272235), and 87.16% (QC 59%) to *Strongyloides stercoralis* (acc. no. KT307990). Similarly, no sequences with high similarity to those generated from the detected *Molineus* spp. (84.6–84.9%; QC 98%; acc. no. JX877696 [*Viannaia minispicula*]), *Heligmosomum* spp. (91.6%; QC 100%; acc. no. MN865448 [*Heligmososmum mixtum*]), and *Porrocaecum* spp. (81.9–82.6%; QC 75–86%; acc. no. AY603532 [*Porrocaecum ensicaudatum*]) were available in GenBank.

The single specimens of *Physaloptera* sp., *Uncinaria* sp., and *Trichuris* sp. were only determined morphologically. Furthermore, a few parasite specimens found in the intestines of three wildcats could not be identified morphologically nor molecularly as these were mainly very small larvae.

The liver fluke *M. bilis* was detected in 2 (2.0%) of the 101 available gallbladders. Molecular species identification revealed 98.3–100% nucleotide identity to published *M. bilis* sequences (QC 100%; acc. nos. KT740966, KT740973). No parasites were found in the liver tissues or bile ducts.

### Parasites of the urinary tract

No parasites were found in the available kidneys of 95 *F. silvestris*. *Capillaria* spp. were present in 77 (77.8%) of the 99 available urinary bladders. Since adult specimens of *C. plica* and *C. feliscati* can only be distinguished morphologically with certainty on the basis of the male posterior ends [14, 27], female specimens, isolated anterior ends, and lysed or damaged worms were summarized as *Capillaria* species. *Capillaria feliscati* was identified in 34 (34.3%) and *C. plica* in 28 (28.3%) urinary bladders. In 11 (11.1%) of these cases, coinfection with both species was proven. In contrast, in 26 (26.3%) further wildcats, species determination was not possible. Of all 538 collected *Capillaria* specimens, 17.7% (95) were identified as *C. feliscati* and 15.6% (84) as *C. plica*.

The intensity of infection with *C. feliscati* and/or *C. plica* was 1–75 (mean 7.0; median 4.0) specimens per wildcat.

Molecular examinations of 11 of these nematodes confirmed the morphological identification. Sequences of *C. plica* were 98.2–100% (QC 100%; acc. no. KC355427) identical to those in GenBank. However, no reference sequence of *C. feliscati* was available, and obtained sequences of *C. feliscati* showed highest identity with an *Aonchotheca erinacei* sequence (86.3–86.8%; QC 95–100%; acc. no. OQ078761), followed by *C. putorii* (85.0–86.3%; QC 94–97%; acc. no. KC355428) and *Capillaria hepatica* (syn. *Calodium hepaticum*; 84.7–85.9%; QC 91–95%, acc. no. MF962896).

The seven detected *C. plica* sequences differed from each other by 0–2.5% (0–6 of 229–331 nucleotides), and the four *C. feliscati* sequences by 0–0.4% (0–1 of 234–332 nucleotides). The difference between the *C. plica* and the *C. feliscati* sequences was 16.5–17.9% (40–57 of 229–331 nucleotides). The different lengths of the sequences obtained (229–332 nucleotides) is due to the fact that two different primer pairs were used to target the COX-1 gene (cf. Table 1), resulting in different amplicon sizes. Thus, the comparisons could only be carried out over the entire length of the shorter sequence, where the query cover was always 100%.

### Parasites of the musculature

Roundworm larvae were detected in 33.0% (37/112; 95% CI 24.4–42.6) of the 112 examined muscle samples, while no wildcat was positive for *Trichinella* larvae. Molecular analysis of one larvae sample resulted in 99.8% identity with a *T. cati* sequence (QC 100%; acc. no. KY003079).

### Statistical analyses

Additional file 2 shows the individual prevalence values of the endoparasite species for the predictor variables that were analyzed in the GLMs. The GLMs for the prevalence of *A. tubaeforme*, *E. multilocularis*, and *M. litteratus* differed significantly from the corresponding null models (Table 4) and indicated that infections with *A. tubaeforme* were significantly more common in subadult (46.7% [7/15]; *P* = 0.042) than in adult (27.8% [10/36]) wildcats and more frequent in summer (87.5% [7/8]) than in winter (5.6% [1/18]; *P* = 0.005). The prevalence of *M. litteratus* was lower in moderately rotten (22.2% [2/9]; *P* = 0.015) than in fresh (82.1% [23/28]) carcasses. In addition, the prevalence of *E. multilocularis* was significantly influenced by sex, with males (29.3% [12/41]) more frequently affected than females (8.8% [3/34]; *P* = 0.050). In contrast, no significant predictors were identified for the remaining parasites, as these GLMs did not differ significantly from the corresponding null models (*H. taeniaeformis*: *χ*^2^ = 17.7, *df* = 11, *P* = 0.090; *C. petrowi*: *χ*^2^ = 13.3, *df* = 11, *P* = 0.274; *Strongyloides* spp.: *χ*^2^ = 15.3, *df* = 11, *P* = 0.168; *C. putorii*: *χ*^2^ = 11.4, *df* = 11, *P* = 0.414; *C. feliscati*/*plica*: *χ*^2^ = 18.2, *df* = 11, *P* = 0.077). Moreover, no significant effect on the sum of co-infections with gastrointestinal parasites was found (*χ*^2^ = 7.9, *df* = 11, *P* = 0.725; Additional file 3).

**Table 4:** Results of GLMs testing the influence of various predictor variables on the prevalence of *Ancylostoma tubaeforme*, *Mesocestoides litteratus*, and *Echinococcus multilocularis* in 75 European wildcats

	*A. tubaeforme*				*M. litteratus*				*E. multilocularis*			
	Estimate	SE	*z*-value	*P*-value	Estimate	SE	*z*-value	*P*-value	Estimate	SE	*z*-value	*P*-value
Intercept	−0.65	0.81	−0.79	0.428	0.86	0.81	1.06	0.290	−2.63	1.23	−2.14	0.032*
Sex (Reference: male)												
Female	−0.56	0.72	−0.78	0.433	−0.73	0.68	−1.07	0.285	−1.59	0.81	−1.96	0.050*
Age (Reference: adult)												
Subadult	1.93	0.95	2.03	0.042*	0.63	0.99	0.64	0.524	1.14	0.93	1.23	0.217
Immature	1.64	0.96	1.71	0.087	−0.11	0.84	−0.13	0.896	1.07	0.87	1.23	0.220
Juvenile	−16.21	1750.26	−0.01	0.993	−2.77	1.51	−1.83	0.067	−18.32	4511.64	0.00	0.997
Nutritional condition (Reference: very good/good)												
Moderate	0.27	1.14	0.23	0.815	0.00	1.09	0.00	0.999	0.75	1.29	0.59	0.558
Very bad/ cachectic	0.75	1.23	0.61	0.543	2.38	1.46	1.63	0.103	−15.10	3479.69	0.00	0.997
Season of finding												
Summer versus spring	2.53	1.33	1.91	0.211	0.16	1.08	0.15	0.999	−16.85	3224.00	−0.01	1.000
Autumn versus spring	−0.77	0.85	−0.91	0.789	1.19	0.90	1.33	0.540	0.93	1.18	0.79	0.833
Winter versus spring	−3.42	1.36	−2.51	0.054	1.48	1.06	1.39	0.503	1.91	1.21	1.58	0.333
Autumn versus summer	−3.30	1.37	−2.40	0.071	1.03	1.08	0.95	0.774	17.77	3224.00	0.01	1.000
Winter versus summer	−5.95	1.82	−3.26	0.006*	1.32	1.22	1.08	0.698	18.76	3224.00	0.01	1.000
Winter versus autumn	−2.65	1.23	−2.15	0.129	0.29	0.97	0.30	0.991	0.98	0.82	1.20	0.575
State of decomposition (Reference: fresh)												
Moderate fresh	−0.17	0.76	−0.23	0.818	−0.01	0.75	−0.02	0.987	0.46	0.78	0.60	0.551
Moderate rotten	−0.17	1.36	−0.12	0.901	−2.87	1.18	−2.44	0.015*	−0.04	1.33	−0.03	0.978

SE: standard error

^*^Significant *P*-values (≤ 0.05)

The full models were significantly different from null models containing only the intercept (*A. tubaeforme*: *χ*^2^ = 31.3, *df* = 11, *P* = 0.001; *M. litteratus*: *χ*^2^ = 22.2, *df* = 11, *P* = 0.023; *E. multilocularis*: *χ*^2^ = 20.7, *df* = 11, *P* = 0.037)

## Discussion

European wildcats can be hosts of many different parasite species, as indicated by previous publications [9–12, 45]. To determine the current and most representative endoparasite status in Germany, 99–104 different organs and 112 pieces of skeletal muscles from a total of 118 European wildcats were screened, providing the first study investigating such a large sample size of wildcats in Germany. Overall, 99.0% of the examined wildcats harbored endoparasites, with infection rates of up to 99.0% in the different organs.

### Endoparasite prevalence and infection intensities

With 95.2% positive wildcats, the prevalence of *T. cati* was the highest among all detected endoparasites, similar to previous studies from Germany with frequencies of 73.3–88.3% [10, 12], while *T. cati* was second most prevalent in Croatia and Greece with 50.0–60.9% positive wildcats [9, 11]. This roundworm is also one of the most common parasites of domestic cats (*Felis catus*) [14], and infects its definitive host by different routes including direct ingestion of infective eggs, ingestion of paratenic hosts (e.g., small rodents), or transmammary transmission [14]. Through these various effective transmission routes, the parasite can spread easily [9]. The *T. cati* infection intensities varied between 1 and 411 specimens per wildcat. Toxocarosis can be particularly dangerous for young domestic cats: High infection intensities can lead to gastrointestinal signs such as diarrhea, vomiting, delayed development, or a distended abdomen. In adult cats, infections are usually inapparent [13]. However, since there are no data on the relationship between infection intensity and pathogenicity of endoparasites in the European wildcat, the intensities have only limited significance for the assessment of wildcat health, all the more so as clinical signs are described for domestic cats and extrapolation to wildcats should be considered with caution. In both this study and the study by Steeb [12], most of the wildcats were in good nutritional condition, which indicates a good general health status, and no correlations of parasite infection with nutritional condition were observed. Interestingly, the present study revealed roundworm larvae also in the skeletal muscles of 33.0% of the animals, which has not previously been described in European wildcats. Molecular analysis of an exemplary muscle sample identified *T. cati*, but it remains unclear whether the other wildcats also harbored *T. cati*, as infections with *Toxocara canis* or *Baylisascaris* spp. larvae also need to be taken into account.

*Hydatigera kamiyai* was the second most common endoparasite, with a prevalence of 84.6%. In other studies, *H. taeniaeformis* was similarly frequent with 53.3–80.5% infected animals [9–12]. Like *T. cati*, *H. taeniaeformis* sensu lato (s.l.) occurs globally. The cryptic species *H. kamiyai* occurs mainly in northern Eurasia with primarily voles (Arviculinae) and *Apodemus* mice as intermediate hosts [25, 46]. Food analyses showed that these small rodents are the main component of the diet of European wildcats in Germany, which favors infections with the tapeworm [3]. Infections with *H. taeniaeformis* s.l. are usually harmless, but heavy infections can lead to intestinal obstruction in domestic cats [13, 14]. The same can be assumed for wildcats, and significant infection intensities of 51 and 53 worms were noted in two animals of the present study.

Prevalence values of the other gastrointestinal parasites vary between different European studies. For *M. litteratus*, it was 69.2% in the present study, similar to the value of 64.9% determined by Steeb [12], but higher than in other studies with 5.9–17.4% [9–11]. There were also differences among the identified species: Krone et al. [10] diagnosed *M. litteratus*, Martinković et al. [11] *Mesocestoides lineatus*, and Steeb [12] and Diakou et al. [9] did not determine the species. Although both species occur in Europe, all ten specimens selected for molecular species identification were genetically identified as *M. litteratus* in the present study. These specimens originated from all regions of Rhineland-Palatinate (except from the area of Taunus), suggesting that *M. litteratus* is at least the predominant species in the sampled German federal state or even the whole country. *Strongyloides* spp. have so far only been detected twice in *F. silvestris* [11, 12] with lower prevalence values than in this study (23.5–28.6% versus 58.7%). The nematodes are very small and thin and can be easily overlooked in examinations at low magnification or slip through sieves larger than 100 µm during sample preparation. Here, *Strongyloides* spp. were mainly found in the contents of the 100-µm sieve and sometimes fewer specimens were additionally in the 50-µm sieve. This could explain the lower detection rate by Steeb [12], where 150-µm sieves were used. For *M. litteratus*, a significant influence of the examination conditions was observed, as they were detected significantly more frequently in fresh than in moderately rotten carcasses. Since the tegument of cestodes has a more fragile tissue structure than the cuticle of nematodes, cestodes are presumably more prone to decomposition so that they could no longer be detected. Even though the statistical analyses only showed this for *M. litteratus*, such an effect can be assumed to some extent for all smaller parasites (e.g., *Strongyloides* spp., *Capillaria* spp., *E. multilocularis*, and *Molineus* spp.), affecting the prevalence and the infection intensity. Regarding pathogenicity, even high infection intensities with *M. litteratus* and *Strongyloides* spp., as in the present study with up to 3926 and 2400 specimens, respectively, have no clinical effects in domestic cats [13, 14].

The prevalence values of *C. petrowi* (37.5%) and *A. tubaeforme* (31.7%) were similar to a study from Greece with 34.8% and 39.1% [9] and higher than in other studies from Germany and Croatia (*C. petrowi* 6.7–11.8% and *A. tubaeforme* 3.9–14.7%) [10–12]. While *C.* *petrowi* causes gastric nodules that are supposed to have no clinical significance in cats [16], *A. tubaeforme* can lead to severe disease in their hosts owing to hematophagia. Infections can cause weight loss, diarrhea, and anemia, among other signs, which can be very dangerous, especially in kittens [13, 14]. In the present study, the highest infection intensity was 135 *A. tubaeforme*. The wildcat host was adult, in a very bad/cachectic nutritional condition, and additionally infected with 3 *H. kamiyai*, 142 *T. cati*, 378 *Strongyloides* spp., and lungworms (31 *Angiostrongylus chabaudi*, 15 *Aelurostrongylus abstrusus*, and 2 *Troglostrongylus brevior* [7]). However, it cannot be estimated whether the poor condition was caused by the high worm burden or vice versa. Regarding factors influencing the prevalence, *A. tubaeforme* was significantly more common in subadult than in adult animals and more abundant in summer than in winter. Younger individuals have an immature immune system, which can explain their higher infection rate [47]. The delay in larval development of *A. tubaeforme* during cold temperatures may cause the seasonal pattern [13].

Mostly localized in the stomachs, *C. putorii* was identified in nearly one-quarter (24.0%) of all wildcats. In Croatia and Germany, *Capillaria* specimens or eggs were found in the gastrointestinal tract or the feces (3.9–50.0%), but the species was not determined [11, 12]. They can cause gastritis and vomiting in domestic cats [13, 48] but do not appear to play a major role in wildcats because all but two of the infections occurred with a maximum of ten detected nematodes.

Besides the present study, *E. multilocularis* and *Molineus* spp. infections in wildcats were also detected by Steeb [12] in Germany. The prevalence of *Molineus* spp. was similar (5.2% versus 5.8%), while *E. multilocularis* was more prevalent in the present study (5.2% versus 18.3%). The detected *E. multilocularis* specimens were very small and often located in the 50-µm sieve, which could explain the higher values compared with Steeb [12], who used a larger sieve (150 µm). The wildcats harbored up to 7506 specimens, but even high infection intensities are not clinically relevant in domestic cats as definitive hosts [13, 14]. Higher infection rates in male than in female hosts are often observed in vertebrates, as noted here for *E. multilocularis*. Possible reasons include immunosuppressive effects of testosterone, behavioral differences, and higher ingestion rates of potentially infected prey [47, 49]. However, this observation could also be a random effect as there are also many reports of female-biased parasitism in literature and influence of sex was not detected for other endoparasite species [49, 50].

The small cestode *T. martis* occurs in mustelids (Mustelidae) and wildcats [13] as definitive hosts. It was one of the rarer parasites, with 9.6% infected animals. Steeb [12] also detected unidentified *Taenia* spp. in some (2.6%) wildcats but suggested that they might be *Taenia pisiformis*.

Only single specimens of *Physaloptera* spp. and *Uncinaria* spp. were detected (1.0% positive samples each). While higher prevalence values of *Physaloptera* spp. were found in Croatia and Greece (11.8–17.4%), there are no reports for *Uncinaria* spp. in European wildcats so far. Normally, the hookworm parasitizes in the small intestine of canids, rarely of felids [13]. The specimen in the present study was found in the stomach, so it was most likely a spurious parasite originating from a mustelid prey species. Although there are cat-specific *Trichuris* spp. (*Trichuris felis*, *Trichuris campanula*, and *Trichuris serrata*), it is more likely that the detected specimen was also a spurious parasite ingested via prey (e.g., *Trichuris muris* with small rodents), as it was detected in the stomach, while *Trichuris* spp. generally parasitize the cecum and colon of their host [14, 51]. *Heligmosomum* spp. and *S. frederici* are typical parasites of voles and *Apodemus* mice, respectively [38, 52]. *Porrocaecum* spp. mainly affect birds, but also mammals, reptiles, and fish [53]. Thus, these species are most likely also spurious parasites, e.g., introduced by prey, rather than actual parasites of *F. silvestris*.

The frequent occurrence of urinary bladder worms (77.8%) was surprising because previous reports are rare. Only in Germany, lower prevalence values of 6.7–39.5% *C. plica*- and *C. feliscati*-positive *F. silvestris* were reported [10, 12]. No difference between the prevalence of *C. plica* and *C. feliscati* was detected in this study (28 versus 34 cases), and coinfections with both species were detected in 14.3% of the 77 infected animals. This is only a minimum value, as not all specimens could be identified to species level. Infections with up to 75 specimens per wildcat were detected. It has been described that *C. plica* are more frequently located in the mucosa and *C. feliscati* on its surface [54]. Both species can cause similarly mild signs such as cystitis and dysuria in *F. catus* [13, 54].

To the best of the authors’ knowledge, *M. bilis* was proven for the first time in a European wildcat in Germany. Hering-Hagenbeck and Schuster [55] found this trematode in 4 of 23 (17.4%) domestic cats in the German state Brandenburg. Other trematodes such as *Alaria alata* or *Opisthorchis felineus* were detected in *F. silvestris* in Croatia and Greece [9, 11].

Like all carnivores, wildcats can become infected with *Trichinella* species. However, *Trichinella* larvae were not detected in this study, but in 5.9% of wildcats in Croatia [11], in 22.5% in Slovenia [56], and 41.3% in Bosnia and Herzegovina [57].

### Molecular endoparasite species determination

The morphological determination of most endoparasite species was confirmed by DNA analysis. *Hydatigera kamiyai* is a new cryptic species belonging to the *H. taeniaeformis* complex and was first described by Lavikainen et al. [25]. The *H. taeniaeformis*-like cestodes found in this study showed highest NAD-1 and SSU rRNA sequence similarities to *H. kamiyai*. They also showed high nucleotide identity with a sequence designated as *T. taeniaeformis*, but this sequence was published prior to the first description of *H. kamiyai* and according to its sequence it also seems to be *H. kamiyai*. With regard to the SSU rRNA gene, sequences were 100% identical to sequences of *H. kamiyai*, but also to a sequence determined to be *H. taeniaeformis*. However, the SSU rRNA differed 9.1–11.0% from two other *H. taeniaeformis* sequences, indicating that the gene region is suitable for distinguishing the two species.

No appropriate sequence was available in GenBank for the molecular identification of *Molineus* species. Thus, species determination was based on morphological criteria only [29]. *Capillaria feliscati* is another nematode for which no molecular confirmation was possible. This hairworm is known from the urinary bladders of cats, but so far it was not certain whether it is a distinct species or identical with *C. plica* [13, 14]. Morphological differentiation between adults can be achieved on the basis of the posterior end of males, as the terminal ala of *C. plica* is triangular, whereas it is rounded in *C. feliscati* [14, 27]. Adult female differentiation is also possible because *C. plica* have a funnel-like vulval appendage that is absent in *C. feliscati*. However, this criterion is much more uncertain than the distinguishing feature of males because sometimes it is difficult to see the appendage of *C. plica* [27]. During this study, specimens with these characteristic criteria were separated and molecular analyses were performed. Nematodes with a caudal triangular ala or vulval appendage, respectively, were identified molecularly as *C. plica* with 98.2–100% identity to published sequences. The sequences obtained from specimens identified morphologically as *C. feliscati* differed from each other by 0–0.4%, but did not show high concordance with any sequences published in GenBank. Their nucleotide difference from the *C. plica* specimens from this study was 16.5–17.9% (QC 100%), which is comparable to distances to other Capillariidae such as *A. erinacei* (13.2–13.7%; QC 95–100%), *C. putorii* (13.7–15.0%; QC 94–97%), and *C. hepatica* (14.1–15.4%; QC 91–97%). These large genetic differences combined with the morphology strongly suggest that *C. plica* and *C. feliscati* are separate species of urinary bladder worms, both of which can infect *F. silvestris*.

### Zoonotic potential and threat from or to domestic cats

The endoparasites detected in this study may not only pose a risk to wildcats. Infections with *E. multilocularis* can cause very severe disease in intermediate and accidental hosts such as humans or even dogs (*Canis lupus familiaris*) and horses (*Equus caballus*) [13]. Fortunately, cats are regarded as poor hosts for this dangerous zoonotic cestode and are less relevant for its spread than red foxes (*Vulpes vulpes*), raccoon dogs (*Nyctereutes procyonoides*), and dogs [58]. The detection of exclusively immature *E. multilocularis* specimens supports this assumption and is consistent with observations by Steeb [12] and Umhang et al. [59]. However, in France, large quantities of *Echinococcus* eggs have been detected in some fecal samples from naturally infected cats, so felids should not be completely disregarded as possible spreaders of *E. multilocularis* [60].

In contrast, felids are the main hosts for *T. cati*, excreting large numbers of viable eggs. In addition, a spillover between wild and domestic cats may occur via paratenic hosts such as small rodents [10] or infective eggs in the environment [34]. It remains unclear whether the wildcat is the original reservoir or if feral or free-ranging domestic cats introduced the parasites into the wildcat population. However, a transmission between both populations is now likely. Aside being a pathogen of felids, *T. cati* is also a zoonotic agent. Ingestion of infectious eggs can lead to visceral or ocular larva migrans, covert toxocarosis, or neurotoxocarosis in humans, particularly in children [61]. With a prevalence of 95.2% in *F. silvestris*, they represent a current reservoir, as *Toxocara* eggs can survive for years [62, 63]. Similarly, *A. tubaeforme* can not only cause disease in felids, but also have zoonotic potential as cutaneous larva migrans, which can cause painful skin lesions in humans [64, 65]. However, compared with other hookworms such as *Ancylostoma caninum* and *Ancylostoma ceylanicum*, *A. tubaeforme* causes cutaneous larva migrans less frequently [65]. Transmission between wild and domestic cats can occur via paratenic hosts, as with *T. cati*. *Capillaria plica* and *C. feliscati* do not have zoonotic potential but should be considered as a differential diagnosis in domestic cats with urinary tract disease.

## Conclusions

Peritoneal organs of up to 104 specimens per organ and skeletal muscle samples of 112 European wildcats were examined with observed infection rates of up to 99.0% per organ. Besides frequent findings of already known common endoparasites of wildcats such as *T. cati* and *H. kamiyai*, *M. bilis* was detected for the first time in a European wildcat in Germany and high prevalence values of *E. multilocularis* as well as *C. plica* and *C. feliscati* were proven. No significant correlations of endoparasite infection status with nutritional status were noted in the studied wildcats, which showed a predominantly good to very good condition. However, there are species such as *T. cati* and *A. tubaeforme* that may cause severe disease in individual hosts, especially in young or weakened animals. Regarding the potential impact on humans, some endoparasite species may pose a risk to them. A spillover to domestic cats may occur, as feral and free-ranging cats invade habitats of wildcats. It remains unclear whether and which parasites may have been introduced into the wildcat population by domestic cats. However, regular deworming of domestic cats is indicated, especially for cats with uncontrolled outdoor access, to minimize the risk also for the pet owner in case of zoonotic parasites.

## Supplementary Information

Additional file 1: Table S1. Comprehensive overview on the key data of the individual German wildcats from which at least three different organs were examined, and respective numbers of endoparasite specimens detected in organs of the peritoneal cavityand the cardiopulmonary system [7] as well as respective number of ectoparasite specimens detected in the hides and ears [8]

Additional file 2: Table S2. Endoparasites prevalence values for the predictor variables in the GLM-analyzed European wildcats

Additional file 3: Table S3. Results of GLM testing the influence of different predictor variables on gastrointestinal parasite species richness of 75 European wildcats

## Data Availability

Data supporting reported results are contained within the article and its additional files. Generated sequences were deposited in GenBank under accession numbers PP812129–PP812131 (*T. cati*), PP796351–PP796355 (*H. kamiyai*, NAD–1), PP808783–PP808784 (*H. kamiyai*, SSU rRNA), PP808791–PP808800 (*M. litteratus*), PP812126–PP812128 (*Strongyloides* spp.), PP840086 (*C. petrowi*), PP812123 (*A. tubaeforme*), PP840083–PP840085 (*Capillaria putorii*), PP796350 (*E. multilocularis*), PP808785–PP808790 (*T. martis*), PP812124–PP812125 (*Molineus* spp.), PP840087–PP840090 (*C. feliscati*), PP840091–PP840097 (*C. plica*), and PP840081–PP840082 (*M. bilis*).

## Abbreviations

Acc. no.: Accession number
BLAST: Basic local alignment search tool
BUND: *Bund fuer Umwelt und Naturschutz Deutschland*
COX-1: Cytochrome c oxidase subunit 1
DNA: Deoxyribonucleic acid
dNTPs: Deoxynucleotide triphosphates
GLM: Generalized linear model
ITS: Internal transcribed spacer
IUCN: International Union for Conservation of Nature
NAD-1: NADH dehydrogenase subunit 1
NCBI: National Center for Biotechnology Information
PCR: Polymerase chain reaction
QC: Query cover
RNA: Ribonucleic acid
rRNA: Ribosomal RNA
SSU rRNA: Small subunit of ribosomal RNA
WOAH: World Organisation for Animal Health

## References

[1] Balzer S, Mölich T, Streif S, Tiesmeyer A, Thein J, Nowak C. Status of wildcats in Germany. Natur und Landschaft. 2018;93:146–52.

[2] Piechocki R. Die Wildkatze *Felis silvestris*. 1st ed. Wittenberg Lutherstadt: Ziemsen; 1990.

[3] Lang J. Die Katze lässt das Mausen nicht - Aktuelle Ergebnisse einer Nahrungsanalyse an Europäischen Wildkatzen aus dem Zentrum ihrer Verbreitung. FELIS Symposium—Der aktuelle Stand der Wildkatzenforschung in Deutschland, 16-17. Oktober 2014, Gießen. 2016.

[4] Bundesnaturschutzgesetz. Bundesnaturschutzgesetz vom 29. Juli 2009 (BGBl. I S. 2542), das zuletzt durch Artikel 5 des Gesetzes vom 3. Juli 2024 (BGBl. 2024 I Nr. 225) geändert worden ist. 2009. https://www.gesetze-im-internet.de/bnatschg_2009/. Accessed 09 Aug 2024.

[5] EEC. Council directive 92/43/EEC of 21 May 1992 on the conservation of natural habitats and of wild fauna and flora. 1992. https://eur-lex.europa.eu/legal-content/EN/TXT/?uri=CELEX%3A01992L0043-20130701. Accessed 26 Sep 2022.

[6] Jerosch S, Götz M. Vergleichende Analysen der Lebensraumnutzung von Wildkatzen (*Felis s. silvestris*) in Wald- und Offenland geprägten Habitaten und Schutzempfehlungen. Beiträge zur Jagd- und Wildforschung. 2015;40:31–44.

[7] Bisterfeld K, Raulf M-K, Waindok P, Springer A, Lang J, Lierz M, et al. Cardio-pulmonary parasites of the European wildcat (*Felis silvestris*) in Germany. Parasit Vectors. 2022;15:452. 36471378 10.1186/s13071-022-05578-z PMC9724372

[8] Bisterfeld K, Raulf MK, Springer A, Lang J, Lierz M, Strube C, et al. Ectoparasites of the European wildcat (*Felis silvestris*) in Germany. Int J Parasitol Parasites Wildl. 2024;25:100977. 39297145 10.1016/j.ijppaw.2024.100977 PMC11407961

[9] Diakou A, Migli D, Dimzas D, Morelli S, Di Cesare A, Youlatos D, et al. Endoparasites of European Wildcats (*Felis silvestris*) in Greece. Pathogens. 2021;10:594. 34068209 10.3390/pathogens10050594 PMC8153176

[10] Krone O, Guminsky O, Meinig H, Herrmann M, Trinzen M, Wibbelt G. Endoparasite spectrum of wild cats (*Felis silvestris* Schreber, 1777) and domestic cats (*Felis catus* L.) from the Eifel, Pfalz region and Saarland, Germany. Eur J Wildl Res. 2008;54:95–100.

[11] Martinković F, Sindičić M, Lučinger S, Štimac I, Bujanić M, Živičnjak T, et al. Endoparasites of wildcats in Croatia. Veterinarski Arhiv. 2017;87:713–29.

[12] Steeb S. Postmortale Untersuchungen an der Europäischen Wildkatze (*Felis silvestris silvestris* SCHREBER, 1777). Germany: Germany Dissertation, Justus-Liebig-University Giessen; 2015.

[13] Deplazes P, Joachim A, Mathis A, Strube C, Taubert A, von Samson-Himmelstjerna G, et al. Parasitologie für die Tiermedizin. 4th ed. Stuttgart: Thieme; 2020.

[14] Bowman DD, Hendrix CM, Lindsay DS, Barr SC. Feline clinical parasitology. 1st ed. Iowa: Wiley; 2002.

[15] Ferguson JA, Woodberry K, Gillin CM, Jackson DH, Sanders JL, Madigan W, et al. *Cylicospirura* species (Nematoda: Spirocercidae) and stomach nodules in cougars (*Puma concolor*) and bobcats (*Lynx rufus*) in Oregon. J Wildl Dis. 2011;47:140–53. 21270003 10.7589/0090-3558-47.1.140

[16] Ibba F, Lepri E, Veronesi F, Di Cesare A, Paltrinieri S. Gastric cylicospirurosis in a domestic cat from Italy. J Feline Med Surg. 2014;16:522–6. 24065709 10.1177/1098612X13505577 PMC11112190

[17] Hrckova G, Miterpakova M, O’Connor A, Snabel V, Olson PD. Molecular and morphological circumscription of *Mesocestoides* tapeworms from red foxes (*Vulpes vulpes*) in central Europe. Parasitology. 2011;138:638–47. 21349216 10.1017/S0031182011000047

[18] Meinig H, Boye P, Dähne M, Hutterer R, Lang J. Rote Liste und Gesamtartenliste der Säugetiere (Mammalia) Deutschlands. Naturschutz und Biologische Vielfalt. 2020;170(2).

[19] Leonhardt I, Stockmann M, Bisterfeld K, Bächlein C, Cocchiararo B, Famira-Parcsetich EM, et al. Wildkatzen-Totfundmonitoring in Rheinland-Pfalz 2018–2020—Sachbericht des Projektes des BUND Rheinland-Pfalz gefördert durch das Ministerium für Umwelt, Energie, Ernährung und Forsten Rheinland-Pfalz (MUEEF) mit den Mitteln aus der AKTION GRÜN. 2021. https://www.bund-rlp.de/fileadmin/rlp/Publikationen/Wildkatzen/Totfundmonitoring_Wildkatze_in_RLP2018-2020_BUND_Sachbericht.pdf.

[20] von Thaden A, Nowak C, Tiesmeyer A, Reiners TE, Alves PC, Lyons LA, et al. Applying genomic data in wildlife monitoring: development guidelines for genotyping degraded samples with reduced single nucleotide polymorphism panels. Mol Ecol Resour. 2020;20:662–80. 10.1111/1755-0998.13136 PMC7199164 31925943

[21] World Organisation for Animal Health Manual of diagnostic tests and vaccines for terrestrial animals 2023 WOAH Terrestrial manual 2023. Manual of diagnostic tests and vaccines for terrestrial animals, vol. 12. Paris: World Organisation for Animal Health; 2023.

[22] Waap H, Gomes J, Nunes T. Parasite communities in stray cat populations from Lisbon. Portugal J Helminthol. 2014;88:389–95. 23719370 10.1017/S0022149X1300031X

[23] Takeuchi-Storm N, Mejer H, Al-Sabi MN, Olsen CS, Thamsborg SM, Enemark HL. Gastrointestinal parasites of cats in Denmark assessed by necropsy and concentration McMaster technique. Vet Parasitol. 2015;214:327–32. 26169220 10.1016/j.vetpar.2015.06.033

[24] European commission. Commission implementing regulation (EU) 2015/1375 of 10 August 2015 laying down specific rules on official controls for *Trichinella* in meat. 2015. https://eur-lex.europa.eu/legal-content/EN/TXT/?uri=celex%3A32015R1375. Accessed 11 Nov 2022.

[25] Lavikainen A, Iwaki T, Haukisalmi V, Konyaev SV, Casiraghi M, Dokuchaev NE, et al. Reappraisal of *Hydatigera taeniaeformis* (Batsch, 1786) (Cestoda: Taeniidae) sensu lato with description of *Hydatigera kamiyai* n. sp. Int J Parasitol. 2016;46:361–74. 26956060 10.1016/j.ijpara.2016.01.009

[26] Boch J, Supperer R, Bauer C. Veterinärmedizinische Parasitologie. 6th ed. Berlin: Parey; 2006.

[27] Butterworth EW, Beverley-Burton M. The taxonomy of *Capillaria* spp (Nematoda: Trichuroidea) in carnivorous mammals from Ontario, Canada. Syst Parasitol. 1980;1:211–36.

[28] Dillard KJ, Saari SA, Anttila M. *Strongyloides stercoralis* infection in a Finnish kennel. Acta Vet Scand. 2007;49:37. 18076758 10.1186/1751-0147-49-37 PMC2225404

[29] Durette-Desset MC, Boomker J, Malan FS. *Molineus cati* n. sp. (Nematoda, Trichostrongylina, Molineoidea), a parasite of feral cats, *Felis catus* Linnaeus, 1758 in South Africa. Onderstepoort J Vet Res. 2000;67:173–7. 11131118

[30] Junker K, Lane EP, McRee AE, Foggin C, van Dyk DS, Mutafchiev Y. Two new species of *Cylicospirura* Vevers, 1922 (Nematoda: Spirocercidae) from carnivores in southern Africa, with validation of the related genera *Gastronodus* Singh, 1934 and *Skrjabinocercina* Matschulsky, 1952. Folia Parasitol (Praha). 2013;60:339–52. 24261135 10.14411/fp.2013.035

[31] Mikaeili F, Mirhendi H, Hosseini M, Asgari Q, Kia EB. *Toxocara* nematodes in stray cats from Shiraz, southern Iran: intensity of infection and molecular identification of the isolates. Iran J Parasitol. 2013;8:593–600. 25516741 PMC4266124

[32] Mordvinov VA, Yurlova NI, Ogorodova LM, Katokhin AV. *Opisthorchis felineus* and *Metorchis bilis* are the main agents of liver fluke infection of humans in Russia. Parasitol Int. 2012;61:25–31. 21840415 10.1016/j.parint.2011.07.021

[33] Sprehn CEW. Lehrbuch der Helminthologie: eine Naturgeschichte der in deutschen Säugetieren und Vögeln schmarotzenden Würmer, unter besonderer Berücksichtigung der Helminthen des Menschen, der Haustiere und wichtigsten Nutztiere. 1st ed. Stuttgart: Borntraeger; 1932.

[34] Strube C, Heuer L, Janecek E. *Toxocara* spp. infections in paratenic hosts. Vet Parasitol. 2013;193:375–89. 23312872 10.1016/j.vetpar.2012.12.033

[35] Veciana M, Chaisiri K, Morand S, Miquel J, Ribas A. New biogeographical and morphological information on *Physaloptera ngoci* Le-Van-Hoa, 1961 (Nematoda: Physalopteridae) in South-east Asian rodents. Parasite. 2013;20:23. 23815881 10.1051/parasite/2013023 PMC3718517

[36] Yamaguti S. Systema helminthum. Volume III. The nematodes of vertebrates. 1st ed. New York City: Interscience; 1961.

[37] Yamaguti S. Systema helminthum. Volume II. The cestodes of vertebrates. 1st ed. New York City: Interscience; 1959.

[38] Stewart A, Lowe A, Smales L, Bajer A, Bradley J, Dwuznik D, et al. Parasitic nematodes of the genus *Syphacia* Seurat, 1916 infecting Muridae in the British Isles, and the peculiar case of *Syphacia frederici*. Parasitology. 2018;145:269–80. 28831960 10.1017/S0031182017001470

[39] Trachsel D, Deplazes P, Mathis A. Identification of taeniid eggs in the faeces from carnivores based on multiplex PCR using targets in mitochondrial DNA. Parasitology. 2007;134:911–20. 17288631 10.1017/S0031182007002235

[40] Armua-Fernandez MT, Nonaka N, Sakurai T, Nakamura S, Gottstein B, Deplazes P, et al. Development of PCR/dot blot assay for specific detection and differentiation of taeniid cestode eggs in canids. Parasitol Int. 2011;60:84–9. 21112414 10.1016/j.parint.2010.11.005

[41] Newton LA, Chilton NB, Beveridge I, Hoste H, Nansen P, Gasser RB. Genetic markers for strongylid nematodes of livestock defined by PCR-based restriction analysis of spacer rDNA. Acta Trop. 1998;69:1–15. 9588237 10.1016/s0001-706x(97)00105-8

[42] Guardone L, Deplazes P, Macchioni F, Magi M, Mathis A. Ribosomal and mitochondrial DNA analysis of Trichuridae nematodes of carnivores and small mammals. Vet Parasitol. 2013;197:364–9. 23920054 10.1016/j.vetpar.2013.06.022

[43] Heneberg P, Sitko J, Bizos J. Integrative taxonomy of central European parasitic flatworms of the family Prosthogonimidae Luhe, 1909 (Trematoda: Plagiorchiida). Parasitol Int. 2015;64:264–73. 25724855 10.1016/j.parint.2015.02.003

[44] R Core Team. R: a language and environment for statistical computing. Vienna: R Foundation for Statistical Computing; 2021.

[45] Napoli E, Anile S, Arrabito C, Scornavacca D, Mazzamuto MV, Gaglio G, et al. Survey on parasitic infections in wildcat (*Felis silvestris silvestris* Schreber, 1777) by scat collection. Parasitol Res. 2016;115:255–61. 26377843 10.1007/s00436-015-4742-2

[46] Miljevic M, Rajicic M, Umhang G, Bajic B, Bjelic Cabrilo O, Budinski I, et al. Cryptic species *Hydatigera kamiyai* and other taeniid metacestodes in the populations of small mammals in Serbia. Parasit Vectors. 2023;16:250. 37491284 10.1186/s13071-023-05879-x PMC10369706

[47] Kołodziej-Sobocińska M. Factors affecting the spread of parasites in populations of wild European terrestrial mammals. Mammal Res. 2019;64:301–18.

[48] Curtsinger DK, Carpenter JL, Turner JL. Gastritis caused by *Aonchotheca putorii* in a domestic cat. J Am Vet Med Assoc. 1993;203:1153–4. 8244862

[49] Klein SL. Hormonal and immunological mechanisms mediating sex differences in parasite infection. Parasite Immunol. 2004;26:247–64. 15541029 10.1111/j.0141-9838.2004.00710.x

[50] Lloyd S. Effect of pregnancy and lactation upon infection. Vet Immunol Immunopathol. 1983;4:153–76. 6191430 10.1016/0165-2427(83)90057-0

[51] Anderson RC. Nematode parasites of vertebrates: their development and transmission. 2nd ed. Wallingford: CABI Publishing; 2000.

[52] Janova E, Skoric M, Heroldova M, Tenora F, Fictum P, Pavlik I. Determinants of the prevalence of *Heligmosomum costellatum* (Heligmosomidae: Trichostrongyloidea) in a common vole population in southern Moravia, Czech Republic. J Helminthol. 2010;84:410–4. 20233485 10.1017/S0022149X1000009X

[53] Li L, Guo YN, Zhang LP. *Porrocaecum parvum* n. sp. and *P. reticulatum* (Linstow, 1899) (Nematoda: Ascaridea) from birds in China. Syst Parasitol. 2015;92:141–9. 26358073 10.1007/s11230-015-9593-9

[54] Bedard C, Desnoyers M, Lavallee MC, Poirier D. *Capillaria* in the bladder of an adult cat. Can Vet J. 2002;43:973–4. 12561694 PMC339923

[55] Hering-Hagenbeck S, Schuster R. A focus of opisthorchiidosis in Germany. Appl Parasitol. 1996;37:260–5. 9060173

[56] Brglez J, Železnik Z. Ein Übersicht über die Parasiten der Wildkatze (*Felis silvestris* Schreber) in Slowenien. Z Jagdwiss. 1976;22:109–12.

[57] Omeragic J, Kapo N, Skapur V, Crnkic C, Goletic S, Softic A, et al. Diversity of *Trichinella* species in carnivores from Bosnia and Herzegovina. BMC Vet Res. 2024;20:117. 38521906 10.1186/s12917-024-03964-6 PMC10960444

[58] Kapel CM, Torgerson PR, Thompson RC, Deplazes P. Reproductive potential of *Echinococcus multilocularis* in experimentally infected foxes, dogs, raccoon dogs and cats. Int J Parasitol. 2006;36:79–86. 16199043 10.1016/j.ijpara.2005.08.012

[59] Umhang G, Forin-Wiart MA, Hormaz V, Caillot C, Boucher JM, Poulle ML, et al. *Echinococcus multilocularis* detection in the intestines and feces of free-ranging domestic cats (*Felis s. catus*) and European wildcats (*Felis s. silvestris*) from northeastern France. Vet Parasitol. 2015;214:75–9. 26206606 10.1016/j.vetpar.2015.06.006

[60] Umhang G, Bastien M, Bastid V, Poulle ML, Boué F. High variability in the number of *E. multilocularis* eggs in cat feces collected in the field. Parasitol Int. 2022;89:102583. 35398276 10.1016/j.parint.2022.102583

[61] Overgaauw PA, van Knapen F. Veterinary and public health aspects of *Toxocara* spp. Vet Parasitol. 2013;193:398–403. 23305972 10.1016/j.vetpar.2012.12.035

[62] Glickman LT, Schantz PM. Epidemiology and pathogenesis of zoonotic toxocariasis. Epidemiol Rev. 1981;3:230–50. 7030762 10.1093/oxfordjournals.epirev.a036235

[63] Jansen J, van Knapen F, Schreurs M, van Wijngaarden T. *Toxocara* ova in parks and sand-boxes in the city of Utrecht. Tijdschr Diergeneeskd. 1993;118:611–4. 8211920

[64] Landmann JK, Prociv P. Experimental human infection with the dog hookworm, *Ancylostoma caninum.* Med J Aust. 2003;178:69–71. 12526725 10.5694/j.1326-5377.2003.tb05222.x

[65] Rodriguez-Morales AJ, González-Leal N, Montes-Montoya MC, Fernández-Espíndola L, Bonilla-Aldana DK, Azeñas- Burgoa JM, et al. Cutaneous larva migrans. Curr Trop Med Rep. 2021;8:190–203.

[66] QGIS Development Team. QGIS Geographic Information System. 2021. Open Source Geospatial Foundation. http://qgis.osgeo.org. Accessed 26 Se 2022.

[67] GeoBasis-DE/Bundesamt für Kartographie und Geodäsie. Verwaltungsgebiete 1: 2 500 000 VG2500. 2022. Data licence Germany—attribution—Version 2.0. https://gdz.bkg.bund.de/index.php/default/open-data/verwaltungsgebiete-1-2-500-000-stand-01-01-vg2500.html. Accessed 26 Sep 2022.

