# Supplementary material for: Endoparasites of peritoneal organs and skeletal muscles of the European wildcat (Felis silvestris) in Germany
Source: Parasit Vectors. 2024 Nov 18;17:473. doi: 10.1186/s13071-024-06571-4 (PMC11575206; doi:10.1186/s13071-024-06571-4)
Supplement: Supplementary file 2 — Additional file 2: Table S2. Endoparasites prevalence values for the predictor variables in the GLM-analyzed European wildcats [file 13071_2024_6571_MOESM2_ESM.docx]

**Table S2:** Endoparasite prevalence values for the predictor variables in the GLM-analysed European wildcats.

|  |  | *A. tubaeforme* | *M. litteratus* | *E. multilocularis* | *H. kamiyai* | *Strongyloides* spp. | *C. petrowi* | *C. putorii* | *C. plica* / *C. feliscati* | |
| --- | --- | --- | --- | --- | --- | --- | --- | --- | --- | --- |
| Predictor variable | N | Positives (%) | Positives (%) | Positives (%) | Positives (%) | Positives (%) | Positives (%) | Positives (%) | N | Positives (%) |
| Sex |  |  |  |  |  |  |  |  |  |  |
| male | 41 | 12 (29.3) | 30 (73.2) | 12 (29.3) | 36 (87.8) | 22 (53.7) | 17 (41.5) | 9 (22.0) | 40 | 33 (82.5) |
| female | 34 | 12 (35.3) | 24 (70.6) | 3 (8.8) | 28 (82.4) | 25 (73.5) | 15 (44.1) | 9 (26.5) | 31 | 25 (80.6) |
| Age |  |  |  |  |  |  |  |  |  |  |
| adult | 36 | 10 (27.8) | 27 (75.0) | 4 (11.1) | 30 (83.3) | 25 (69.4) | 14 (38.9) | 8 (22.2) | 34 | 30 (88.2) |
| subadult | 15 | 7 (46.7) | 12 (80.0) | 5 (33.3) | 14 (93.3) | 8 (53.3) | 10 (66.7) | 3 (20.0) | 13 | 10 (76.9) |
| immature | 19 | 7 (36.8) | 13 (68.4) | 6 (31.6) | 16 (84.2) | 11 (57.9) | 6 (31.6) | 6 (31.6) | 19 | 16 (84.2) |
| juvenile | 5 | 0 (0.0) | 2 (40.0) | 0 (0.0) | 4 (80.0) | 3 (60.0) | 2 (40.0) | 1 (20.0) | 5 | 2 (40.0) |
| Nutritional condition |  |  |  |  |  |  |  |  |  |  |
| very good/good | 57 | 15 (26.3) | 44 (77.2) | 13 (22.8) | 48 (84.2) | 38 (66.7) | 21 (36.8) | 15 (26.3) | 53 | 45 (84.9) |
| moderate | 11 | 5 (45.5) | 4 (36.4) | 2 (18.2) | 10 (90.9) | 3 (27.3) | 5 (45.5) | 2 (18.2) | 12 | 9 (75.0) |
| very bad/cachectic | 7 | 4 (57.1) | 6 (85.7) | 0 (0.0) | 6 (85.7) | 6 (85.7) | 6 (85.7) | 1 (14.3) | 6 | 4 (66.7) |
| Season of finding |  |  |  |  |  |  |  |  |  |  |
| spring | 17 | 8 (47.1) | 10 (58.8) | 2 (11.8) | 12 (70.6) | 11 (64.7) | 9 (52.9) | 3 (17.6) | 16 | 16 (100) |
| summer | 8 | 7 (87.5) | 5 (62.5) | 0 (0.0) | 6 (75.0) | 4 (50.0) | 4 (50.0) | 0 (0.0) | 7 | 4 (57.1) |
| autumn | 32 | 8 (25.0) | 24 (75.0) | 5 (15.6) | 30 (93.8) | 20 (62.5) | 10 (31.3) | 9 (28.1) | 31 | 24 (77.4) |
| winter | 18 | 1 (5.6) | 15 (83.3) | 8 (44.4) | 16 (88.9) | 12 (66.7) | 9 (50.0) | 6 (33.3) | 17 | 14 (82.4) |
| State of decomposition |  |  |  |  |  |  |  |  |  |  |
| fresh | 28 | 8 (28.6) | 23 (82.1) | 5 (17.9) | 26 (92.9) | 18 (64.3) | 12 (42.9) | 4 (14.3) | 25 | 19 (76.0) |
| moderate fresh | 38 | 11 (28.9) | 29 (76.3) | 8 (21.1) | 31 (81.6) | 26 (68.4) | 15 (39.5) | 11 (28.9) | 37 | 31 (83.8) |
| moderate rotten | 9 | 5 (55.6) | 2 (22.2) | 2 (22.2) | 7 (77.8) | 3 (33.3) | 5 (55.6) | 3 (33.3) | 9 | 8 (88.9) |
