# Supplementary material for: Endoparasites of peritoneal organs and skeletal muscles of the European wildcat (Felis silvestris) in Germany
Source: Parasit Vectors. 2024 Nov 18;17:473. doi: 10.1186/s13071-024-06571-4 (PMC11575206; doi:10.1186/s13071-024-06571-4)
Supplement: Supplementary file 3 — Additional file 3: Table S3. Results of GLM testing the influence of different predictor variables on gastrointestinal parasite species richness of 75 European wildcats [file 13071_2024_6571_MOESM3_ESM.docx]

**Table S3:** Results of GLM testing the influence of different predictor variables on gastrointestinal parasite species richness of 75 European wildcats.

|  | Estimate | Std. error | *z*-value | *P*-value | |
| --- | --- | --- | --- | --- | --- |
| Intercept | 1.341 | 0.163 | 8.232 | **<0.001** | |
| Sex (Reference: male) |  |  |  |  | |
| female | -0.038 | 0.118 | -0.319 | 0.749 | |
| Age (Reference: adult) | | | | |  |
| subadult | 0.202 | 0.151 | 1.337 | 0.181 | |
| immature | 0.127 | 0.145 | 0.880 | 0.379 | |
| juvenile | -0.397 | 0.317 | -1.254 | 0.210 | |
| Nutritional condition (Reference: very good/good) | | | | |  |
| moderate | -0.013 | 0.220 | -0.057 | 0.955 | |
| very bad/cachectic | 0.319 | 0.217 | 1.470 | 0.142 | |
| Season of finding |  |  |  |  | |
| summer vs. spring | 0.055 | 0.213 | 0.260 | 0.994 | |
| autumn vs. spring | 0.179 | 0.171 | 1.049 | 0.715 | |
| winter vs. spring | 0.165 | 0.187 | 0.881 | 0.811 | |
| autumn vs. summer | 0.123 | 0.218 | 0.565 | 0.941 | |
| winter vs. summer | 0.110 | 0.230 | 0.476 | 0.963 | |
| winter vs. autumn | -0.014 | 0.149 | -0.094 | 1.000 | |
| State of decomposition (Reference: fresh) | | | | |  |
| moderate fresh | -0.018 | 0.125 | -0.147 | 0.883 | |
| moderate rotten | -0.120 | 0.220 | -0.546 | 0.585 | |

Std. error: standard error

Significant *P*-values (≤ 0.05) are printed in bold.

The full model was not significantly different from a null model containing only the intercept (χ^2^ = 7.9, Df = 11, *P* = 0.725).
